# The effect of nodal connectivity and strut density within stochastic titanium scaffolds on osteogenesis

**DOI:** 10.3389/fbioe.2023.1305936

**Published:** 2023-11-29

**Authors:** Stylianos Kechagias, Konstantinos Theodoridis, Joseph Broomfield, Kenny Malpartida-Cardenas, Ruth Reid, Pantelis Georgiou, Richard J. van Arkel, Jonathan R. T. Jeffers

**Affiliations:** ^1^ Department of Mechanical Engineering, Imperial College London, London, United Kingdom; ^2^ Centre for Bio Inspired Technology, Department of Electrical and Electronic Engineering, Imperial College London, London, United Kingdom; ^3^ Department of Surgery and Cancer, Imperial College London, London, United Kingdom; ^4^ Department of Infectious Disease, Imperial College London, London, United Kingdom

**Keywords:** additive manufacturing, orthopaedic implants, bone scaffolds, trabecular bone, 3D cell culture, osteoblast differentiation

## Abstract

Modern orthopaedic implants use lattice structures that act as 3D scaffolds to enhance bone growth into and around implants. Stochastic scaffolds are of particular interest as they mimic the architecture of trabecular bone and can combine isotropic properties and adjustable structure. The existing research mainly concentrates on controlling the mechanical and biological performance of periodic lattices by adjusting pore size and shape. Still, less is known on how we can control the performance of stochastic lattices through their design parameters: nodal connectivity, strut density and strut thickness. To elucidate this, four lattice structures were evaluated with varied strut densities and connectivity, hence different local geometry and mechanical properties: low apparent modulus, high apparent modulus, and two with near-identical modulus. Pre-osteoblast murine cells were seeded on scaffolds and cultured *in vitro* for 28 days. Cell adhesion, proliferation and differentiation were evaluated. Additionally, the expression levels of key osteogenic biomarkers were used to assess the effect of each design parameter on the quality of newly formed tissue. The main finding was that increasing connectivity increased the rate of osteoblast maturation, tissue formation and mineralisation. In detail, doubling the connectivity, over fixed strut density, increased collagen type-I by 140%, increased osteopontin by 130% and osteocalcin by 110%. This was attributed to the increased number of acute angles formed by the numerous connected struts, which facilitated the organization of cells and accelerated the cell cycle. Overall, increasing connectivity and adjusting strut density is a novel technique to design stochastic structures which combine a broad range of biomimetic properties and rapid ossification.

## 1 Introduction

Additive manufacturing has revolutionised the production of modern orthopaedic implants that partially or fully consist of lattice structures. Lattice structures are 3D porous networks formed by interconnected struts or sheets with fully controllable micro-architecture and macro-properties. A lattice structure on an implant serves as a scaffold that mimics the network of trabecular bone enabling cells from the host bone to migrate inside it and form new bone extracellular matrix (ECM) directly on implant’s surface–a process termed as osseointegration (Albrektsson and Johansson, 2001). Bone, however, is a dynamic tissue that continually remodels in response to the physiological loading that arises from daily activity. Thus, an osseointragative implant should not only provide a biomimetic environment for bone regeneration but should also aid bone maintain its homeostasis.

The stiffness of trabecular bone typically ranges between 0.02 and 5 GPa depending on age, health condition and anatomical site (Morgan et al., 2003). Lattice-based implants made of much stiffer materials, such as titanium and cobalt-chrome alloys, can be designed to match this range of stiffness and allow bone to experience more physiological loading upon implantation by preventing the “stress shielding” phenomenon (Joshi et al., 2000; Hadjidakis and Androulakis, 2006). However, reduced lattice stiffness is accompanied by drastic reduction of its mechanical strength. The mechanical properties of a lattice depend on its structural material, its porosity and its topology (pores’ shape and strut connectivity) (Kechagias et al., 2022). Porosity which is controlled by the pore sizing and strut thickness, has a negative correlation to mechanical properties, while increased connectivity results to more rigid structures with higher fatigue strength (Ashby, 2006; Kechagias et al., 2022). Therefore, the design of lattice structures for bone replacement implants remains a challenge due to the simultaneous need of micro-architectures that provide cues for tissue growth and macro-properties that offer a stimulative environment with high load-bearing capabilities.

From a biological perspective, scaffolds should have interconnected open pores with size and shape that favours bone tissue formation and maturation for superior implant-to-bone fixation (Chen et al., 2020). Small pores increase scaffold’s specific surface area that benefits initially cell adhesion and later cells communication towards rapid differentiation, tissue formation and mineralisation (Van Bael et al., 2012; Torres-Sanchez et al., 2022). Large pores, on the other hand, result in high fluid permeability allowing deep cell migration, oxygen and nutrients transportation, aiding tissue in-growth and vascularisation in large-sized implants (Van Bael et al., 2012; Taniguchi et al., 2016; Shah et al., 2019). Pore sizes between 300 and 1,000 μm satisfy these criteria while avoiding phenomena such as precocious pore occlusion due to very small pores (Van Bael et al., 2012) or poor tissue formation and mineralisation due to very large pores (Frosch et al., 2002). Many *in vitro* and *in vivo* studies conclude that pore sizes of approximately 600 μm optimise scaffold biological properties, yet holistic review of literature suggests that an optimal pore size highly depends on pore geometry and topology (Karageorgiou and Kaplan, 2005; Wang et al., 2017; Deering and Grandfield, 2021).

Lattice topologies where struts form acute angles (i.e., triangulated pores) have found to accelerate cell differentiation compared to topologies with obtuse-angled or circular-shaped pores (Van Bael et al., 2012; Markhoff et al., 2015; Man et al., 2021). While studies in channels of different shapes report that small radius of curvature and the presence of concavities greatly accelerate tissue growth (Rumpler et al., 2008; Bidan et al., 2013). This is typically attributed to the curvature-driven tissue formation, where tissue preferably grows in concave regions of 3D environments like pore corners (Rumpler et al., 2008). ECM bridges the corners to increase the radius of curvature and progressively occludes the pores as more cells sense and bind to the ECM (Callens et al., 2020), with smaller pores accelerating the rate of tissue formation (Bidan et al., 2013; Knychala et al., 2013; Buenzli et al., 2020). Cell proliferation and differentiation are driven by local forces that cells sense from substrate geometry, thus scaffold design has always focused on tuning pore size and geometry to provide physical cues for beneficial cell motility and communication (Vogel and Sheetz, 2006; Nune et al., 2016).

Lattice micro-architectures can be either periodic (pores with regular shape) or stochastic (pores with random shapes). Most studies have employed periodic lattices, however stochastic lattices better mimic the architecture of trabecular bone and have been shown to favour cellular behaviour over periodic lattices of similar pore size or surface area (Wang et al., 2021; Liang et al., 2022; Torres-Sanchez et al., 2022). From an engineering point of view, stochastic designs benefit the fabrication of lattice-based parts with irregular or curved geometry (Emmelmann et al., 2011) and can be designed with more isotropic mechanical properties (Hossain et al., 2021a). Limited data exist regarding how stochastic lattices affect osseointegration, where existing studies have focused solely on the influence of pore size (Wang et al., 2020; Jiao et al., 2023). Yet, pore size typically varies inside such heterogeneous structures compared to periodic lattices that demonstrate a more confined distribution (Liang et al., 2019), thus pore size might not be adequate for controlling cellular behaviour in stochastic designs.

We have previously shown that stochastic lattices can be fully defined using three controllable design parameters: nodal connectivity, strut density and strut thickness (Kechagias et al., 2022). Each has positive correlation with stiffness and strength, meaning that different parameter combinations can result in lattices with different local geometries yet equivalent mechanical properties. This gives a new element of design freedom, enabling simultaneous design for mechanical properties and biological performance.

Pore size and spatial concavities standout as key factors that benefit bone tissue formation. Thus, we hypothesized that high connectivity lattices could combine biomimetic stiffness and high mechanical performance together with suitable pore sizes and multiple corners to help mature bone regeneration inside porous implants. This work will employ lattices designed with different connectivity levels and strut densities to examine how the junction of these parameters affect *in vitro* cell proliferation, differentiation, and tissue formation. Interpretation of our results should disclose a novel perception for designing biomimetic bone scaffolds as next-generation bone implants.

## 2 Materials and methods

### 2.1 Scaffolds design and manufacturing

**FIGURE 1:**
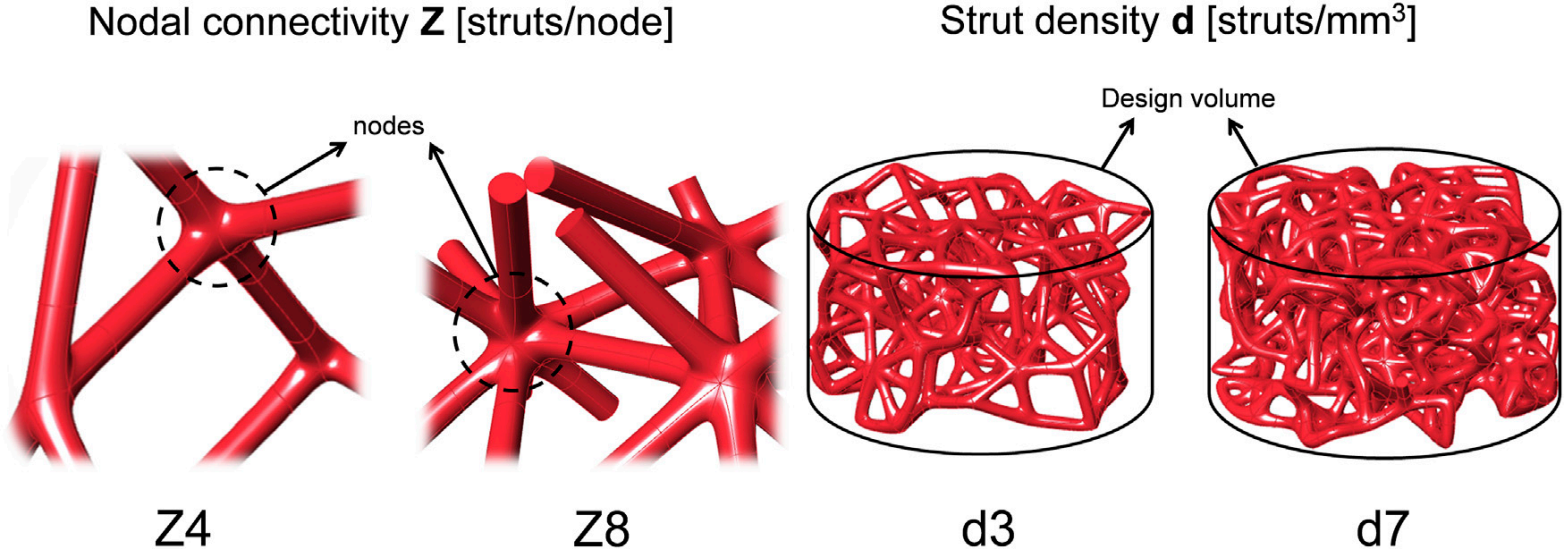
Sketches of the design parameters. Scaffolds were designed by combining two levels of nodal connectivity Z and two levels of strut density d.

Stochastic lattice structures were designed using Rhinoceros 3D Software (Robert McNeel and Associates, United States) as line geometries which enables higher control of the fabrication process (Ghouse et al., 2017). Ø12 × 6 mm cylinders were filled with pseudo-randomly distributed nodal points. Zero thickness lines then connected all nodes to each other, leading to the desired nodal connectivity of each lattice. Lines with inclination angle <25° with respect to the building bed were adapted to 25° by kinking their midpoints, to overcome printing limitations (Hossain et al., 2021a). Four scaffold designs were created using two connectivity levels ( Z = 4 and Z = 8) and two values of strut density (i.e., number of struts inside the design volume, here d = 3 and d = 7 struts/mm^3^) as described in Kechagias et al. (2022) and shown in Figure 1–hereafter they will be referred as Z4-d3, Z4-d7, Z8-d3, and Z8-d7.

Scaffolds were fabricated using an AM250 powder bed fusion system (Renishaw plc, United Kingdom) with commercially pure titanium powder (ASTM B348 Grade 2, Ø15-45 μm, D50: 27 μm, Carpenter Additive, US). An in-house software was used to slice the line geometries and assign tailored laser parameters depending on struts’ angles in respect to the building bed to produce a uniform strut thickness of 230 μm as described and assessed in (Ghouse et al., 2017; Kechagias et al., 2022). Detailed laser parameters are provided in the Supplementary Material (Supplementary Table S1).

A total of 120 scaffolds were fabricated (*n* = 30/design). Specimens were removed from the building bed by wire electro discharge machining (AQ400L, Sodick Inc., Japan) using water as dielectric fluid, 30 V and feet rate of 0.4 mm/min. Scaffolds were cleaned ultrasonically in acetone, left immersed in 4 M NaOH for 24 h and then washed with phosphate buffered saline (PBS) to stabilize their pH value to 7.4. NaOH was used to remove any contaminants in scaffolds and increase titanium surface hydrophilicity (Kim et al., 2013). Difference in the surface topography due to alkali treatment with NaOH is shown in Supplementary Figure S1.

Additionally, individual (12-mm long) struts were printed at different angles with respect to the building bed using the same laser parameters and their surface roughness was measured using a digital microscope (RH200, Hirox, Japan). For this purpose, the heights along a 75-μm long profile line were acquired using a 0.55 μm linear step, 0.39 µm resolution and ×400 dry objective lens. Three unique struts were used per building angle and the arithmetic average roughness (Ra) for two profiles per strut (one at the upward- and downward-facing surface as printed) was recorded.

### 2.2 Scaffolds’ micro-architecture characterisation

After cleaning, all samples were weighted with an analytical scale and measured with micro-callipers to define their porosity through the gravimetric method:

$$Porosity=1-\frac{\rho_{a}}{\rho_{s}}$$

Where, $\rho_{a}$ corresponds to scaffold’s apparent density (the ratio of specimen’s mass to measured volume) and $\rho_{s}$ corresponds to the density of solid pure titanium ( $\rho_{s}$ = 4.51 g/cm^3^).

Micro-CT scans were performed using an Xradia 510 Versa (Carl Zeiss AG, Germany) to measure scaffolds’ local geometry. One specimen per design was scanned using 80kV, 5 s exposure, 2041 projections and 12 μm pixel size. CTAn (Bruker Ltd., United Kingdom) was used to measure mean pore size (taken as the trabeculae separation (Hildebrand and Ruegsegger, 1997)), degree of anisotropy and the surface area of each scaffold using the reconstructed scanned data.

Lattice specimens designed and printed with the same parameters, but with larger dimensions (Ø13 mm × 21 mm) according to mechanical testing standards (International Organization for Standardization, 2011) to eliminate size effects (Tekogu et al., 2011), have been assessed in a previous study (Kechagias et al., 2022), with results provided in Table 1.

**TABLE 1:** Measured properties and feature sizes of scaffolds designed with different connectivity Z and strut density d, but fixed strut thickness of 230 μm * Data were retrieved from (Kechagias et al, 2022). ** Values rounded to nearest 10.

	Design			
Property and feature sizes	Z4–d3	Z4–d7	Z8–d3	Z8–d7
Porosity [%] (*n* = 30)	91.6 ± 0.1	86.4 ± 0.4	87.9 ± 0.3	81.3 ± 0.5
Elastic modulus [MPa] (*n* = 5) *	140.3 ± 11.0	605.0 ± 21.1	711.1 ± 42.5	2149.2 ± 149.9
Ultimate strength [MPa] (*n* = 5) *	2.2 ± 0.1	8.5 ± 0.2	8.1 ± 0.3	22.2 ± 0.6
Node density [nodes/mm^3^]	1.6	3.4	0.7	1.7
Degree of Anisotropy	1.10	1.05	1.08	1.12
Pore size [µm]**	1,040 ± 310	750 ± 210	880 ± 370	680 ± 270
Strut length [µm]**	940 ± 220	720 ± 160	1,320 ± 230	990 ± 180
Surface area [mm^2^]	1,354.9	1869.0	2108.9	2392.3

### 2.3 Cell expansion and seeding

Pre-osteoblast cells (MC3T3-E1 Subclone 4, American Type Culture Collection, USA) were expanded with complete culture medium containing of MEM (Minimum Essential Medium, Gibco™, Thermo Fisher Scientific, United States), 10% FBS (Gibco™, Thermo Fisher Scientific, United States), and 1% antimicrobial solution (Penicillin-Streptomycin-Amphotericin B, MP Biomedicals, Gibco™, Thermo Fisher Scientific, United States) till Passage 4. Prior cell seeding, scaffolds were autoclaved and immersed in culture medium and incubated (37°C, 5%CO_2_ and 21%O_2_) for at least 2 h to allow for surface protein adsorption (Wilson et al., 2005). Pre-wetted scaffolds were placed in 24-well plates for cell suspension. Cells were counted using a Neubauer chamber, and a total of 1 × 10^6^ cells/scaffold were suspended on the scaffolds, half on the top and half on the bottom surface. After cell suspension on each surface, scaffolds were incubated for 1 h at 37°C, 5%CO_2_ and 21%O_2_, to allow cell attachment within the porous structures. Seeded scaffolds were then transferred to 12-well plates and filled with 5 mL of complete culture medium. Cell seeded scaffolds were cultured for a total of 28 days. Media changes were performed every 3 or 7 days as needed.

### 2.4 Biological assays

#### 2.4.1 Cell adhesion and proliferation rate

A high-resolution sensitivity kit (CCK-8, Enzo Life Sciences, United States) was used to assess cell adhesion within all scaffold designs, 24 h after seeding. Cell seeded scaffolds were transferred to 24-well plates and incubated for 4 h with 0.8 mL of fresh culture medium and 80 µL of CCK-8 solution. Stained media were then added to a 96-well plate and the absorbance was measured at 450 nm using a microplate reader (CLARIOstar Plus, BMG LABTECH, Germany). The assay was also performed using the initial well plates (where the same scaffolds had been seeded) to account for the cells that did not adhere to the scaffolds to estimate cell seeding efficiency.

Cell proliferation rate on scaffolds was evaluated by measuring cellular metabolic activity from the beginning of the experiment and for the next 15 days. For that purpose, 10% v/v AlamarBlue (Invitrogen, USA) was added to each well of all scaffold designs and incubated for 4 h. Stained media from the wells were replaced with fresh culture media to continue their culture, given that AlamarBlue is a non-toxic reagent for cells. Stain media were used to measure fluorescent intensity at 560 nm excitation and 600 nm emission. This measurement was repeated every 3 days.

#### 2.4.2 Collagen content

Newly formed ECM was evaluated for collagen concentration at days 7 and 28 using Picrosirius Red staining (1 mg/mL Sirius Red in saturated Picric acid). Scaffolds were initially stored in 10% neutral buffered formalin, cleaned with PBS and immersed in Picrosirius Red overnight. All scaffolds were then washed with distilled water to remove any unbound dye and dried at 37°C. Collagen content was quantified using colorimetry. Bounded dye was removed by immersing scaffolds in 0.1 mol/L NaOH in PBS and incubating for 45 min with 30 rpm shaking. Afterwards, the absorbance of the dissolved dye was measured at 550 nm using a microplate reader.

#### 2.4.3 Pre-osteoblasts maturation

Pre-osteoblasts’ maturation was assessed by measuring the expression levels of key biomarkers linked to osteogenic differentiation.

Alkaline phosphatase activity, which is considered an early marker for osteogenesis, was measured through supernatants collected from all scaffolds at days 7, 14, 21 and 28 using an alkaline phosphatase detection kit (MilliporeSigma, United States). Additionally, the expression levels of collagen type-I (the most abundant collagen type in bone tissue), osteopontin and osteocalcin (bone-specific ECM proteins) were measured at days 7 and 28 through quantitative real-time Polymerase Chain Reaction (RT-qPCR). For the RT-qPCR analysis, cells were extracted from scaffolds using Trypsin-EDTA solution and stored in RNAlater™ Stabilization Solution (Thermofisher, USA). Nucleic acid extraction was performed using the RNeasy kit (QIAGEN, Germany) and the GoTaq 1-step RT-qPCR kit (Promega United Kingdom Ltd., United Kingdom) was used for the analysis. The PCR reaction methodology is described in detail in the Supplementary Material. The sequences of the selected qPCR primers are shown in Table 2. The relative expression levels of the genes of interest compared to untreated samples were determined and normalised to the expression of the reference gene (β-actin) using a modified ΔΔCt method.

**TABLE 2:** qPCR primer sequences for multiple RNA targets.

Target	Forward 5’ → 3′	Reverse 5’ → 3′	Ref
Collagen Type-I	CCT​CAG​GGT​ATT​GCT​GGA​CAA​C	CAG​AAG​GAC​CTT​GTT​TGC​CAG​G	He et al. (2021)
Osteopontin	CCTCCCGGTGAAAGTGAC	CTGTGGCGCAAGGAGATT	Liang and Yu (2018)
Osteocalcin	TGCTTGTGACGAGCTATCAR	GAG​GAC​AGG​GAG​GAT​CAA​GT	Xu et al. (2020)
β-actin	GGG​TCA​GAA​GGA​CTC​CTA​TG	GGT​CTC​AAA​CAT​GAT​CTG​GG	Liang and Yu (2018)

### 2.5 Imaging and image processing

#### 2.5.1 Scanning electron microscopy (SEM)

SEM was used to observe the morphology of cells and formed ECM in all scaffold designs at days 7, 14, 21 and 28. Cell-seeded scaffolds were fixed in 10% neutral buffered formalin, and prior imaging, they were cleaned with PBS and dehydrated through ethanol series (30%, 50%, 70%, 96% and 100% ethanol dilution), air-dried for 24 h and were sputter-coated by a 15-nm-thick chromium layer. SEM was performed using a Mira microscope (TESCAN, Czech Republic) at 10keV and 1 nA.

#### 2.5.2 Fluorescence microscopy

##### 2.5.2.1 Cell viability

A viability/cytotoxicity (live/dead) assay kit (Biotium, USA) was used to stain live and dead cells in one sample per design at days 7, 14, 21 and 28 of culture. The kit employed calcein AM and Ethidium Homodimer III dyes for staining live and dead cells, respectively. Samples were imaged in a SP8 confocal microscope (Leica, Germany) using 496 and 514 nm laser excitation.

##### 2.5.2.2 Mineralised matrix labelling

To assess mineralised ECM regions, a fluorochrome (Calcein, Sigma-Aldrich, USA) was added to the media on day 7, 14, 21 and 28 at a concentration of 50 μg/mL and replaced with fresh media after 24 h. Scaffolds were imaged in a Thunder widefield microscope (Leica, Germany) using 519 nm laser excitation and 535 nm emission, and the acquired images were post-processed using the microscope’s software. Calcein dye is widely used to label bone tissue as it binds to calcium crystals in osteoprogenitor 2D and 3D cell cultures (Hale et al., 2000), thus fluorometric analysis was used to observe the progress of ECM mineralisation.

### 2.6 Statistical analysis

Differences between measured values were assessed using either a 2-way or 3-way analysis of variance (ANOVA) followed by Tukey’s *post hoc* test using GraphPad Prism (GraphPad Software Inc., United States). Connectivity, strut density and day of culture were the independent variables depending on the assay and the significance level was set to *α* = 0.05. Data are presented as mean ± standard deviation with n = 3, unless otherwise stated. In figures (*) indicates *p* < 0.05, (**) indicates *p* < 0.01 (***) indicates *p* < 0.001, and no notation indicates that no difference was found (*p* > 0.05).

## 3 Results

### 3.1 Scaffolds characterisation

The structural and mechanical characteristics of the lattice designs are summarised in Table 1. High connectivity with high strut density (Z8-d7) led to high mechanical properties and low porosity, the low-low (Z4-d3) combination led to low mechanical properties and high porosity, and the two high-low combinations (Z4-d7 and Z8-d3) resulted in similar porosity and mechanical properties. For all samples the porosity fell within the range associate with trabecular bone (typically 70%–90%) (Wang et al., 2017). Increasing Z or d resulted in less porous structures with smaller pore sizes and higher surface area. The high connectivity structures (Z8-d3 and Z8-d7) exhibited the highest surface area which can be attributed to their longer struts (40% increased strut length for fixed strut density) and bulgier nodes (the Z8 nodes had ∼2.5 times higher surface area than the Z4 nodes, see Supplementary Figure S2).

**FIGURE 2:**
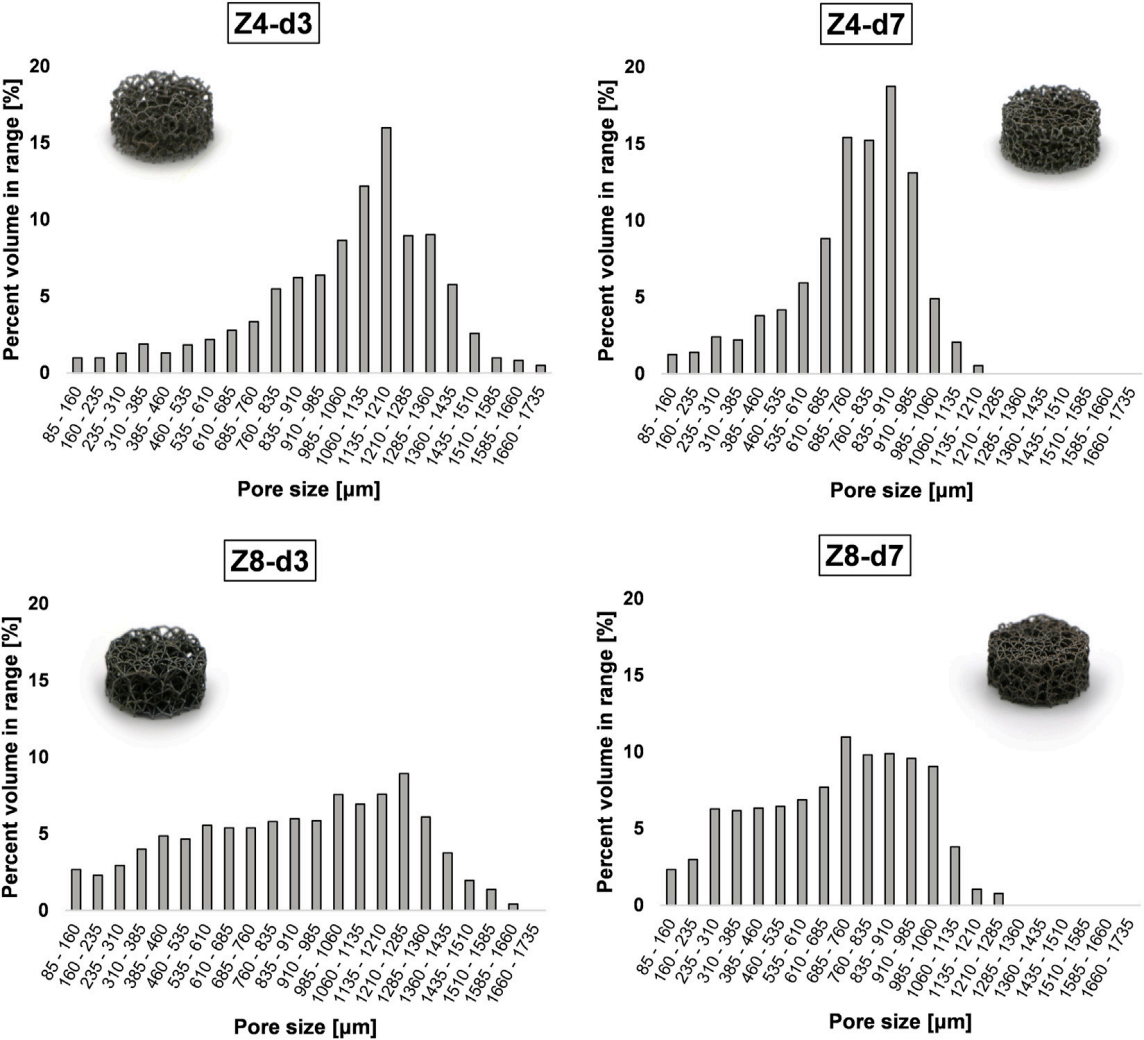
Pore size distribution in the stochastic lattice structures. Increasing connectivity Z appears to increase the spread of pore size inside the structure.

Pore size demonstrated a more confined distribution in low connectivity (Z = 4) scaffolds and distinctly greater variance in high connectivity (Z = 8) scaffolds (see Figure 2). This is because high connectivity results in higher spatial tessellation, introducing multiple corners around the nodes and multiple pores inside the structure. Nevertheless, structures followed a pseudo-random architecture with similar strut orientation in space leading to similar degrees of anisotropy (Hossain et al., 2021a). In addition, structures employed struts in all directions resulting in a degree of anisotropy near 1 (i.e., isotropic material). While trabecular bone is a highly anisotropic material where the degree of anisotropy ranges from 1.1 to ≈5 (Kersh et al., 2013), having isotropic properties in a lattice is important from a design perspective as it allows better prediction of lattice behaviour *in situ*.

**FIGURE 3:**
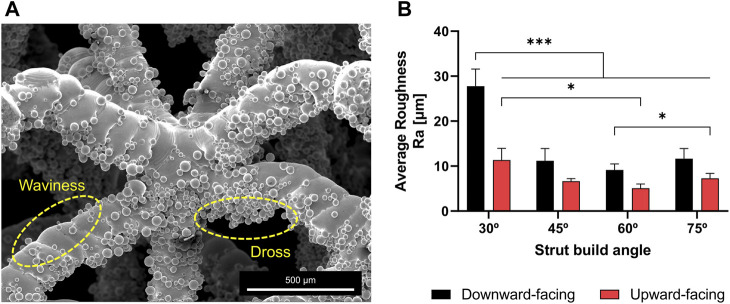
**(A)** SEM images demonstrating typical forms of surface roughness in struts fabricated using powder bed fusion. Numerous semi-sintered particles are stand out from strut surface and around the nodes **(B)** The arithmetic average roughness of the upward and downward facing surfaces of struts built at different angles.

The physical samples exhibited typical forms of roughness found in additively manufactured overhanging surfaces (such as struts) as illustrated in Figure 3A. Surface roughness originated from semi-sintered powder particles and dross formation at the downward-facing struts’ surface due to overheating as well as waviness at the upward-facing struts’ surfaces due to the overlap of consecutive melt-pools. These micro-scale features were able to be quantified using a microscope’s 3D optical profilometer. As shown in Figure 3B, the average roughness (Ra) was found to range between 5 and 30 μm. Ra values tend to increase with decreasing build angle, while the downward-facing surface of struts is always rougher than the upward-facing surface due to higher heat accumulation.

### 3.2 Cell adhesion and proliferation

**FIGURE 4:**
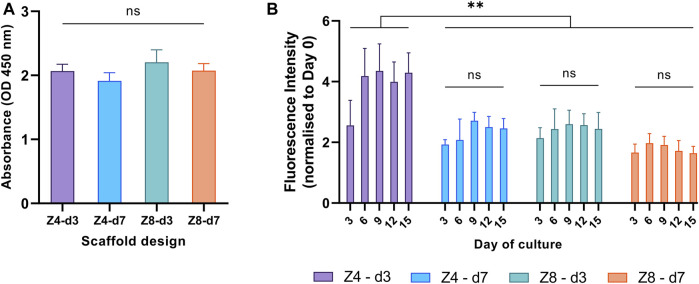
Cellular metabolic activity was used to compare **(A)** cell adhesion in the first 24 h and **(B)** proliferation rate over the first 15 days between different designs. Each column corresponds to the mean value of *n* = 3. ANOVA results are provided in Supplementary Material (Supplementary Tables S2, S3). The main effect of day (*p* < 0.001) is not highlighted.

All samples achieved similar metabolic activity in the first 24 h (Figure 4A), indicating that cell seeding and cell adhesion was equivalent among all scaffold designs. Cell seeding efficiency was estimated to approximately 85% for all scaffolds.

To investigate the proliferation progress on all scaffold designs, from the beginning of the experiment till the 15th day, fluorescent intensity values were measured every 3 days and all measurements from the third day and afterwards were normalised to day 0 (Figure 4B). Cells proliferated on all scaffold designs, as expected. The proliferation rate depended on the culture day (*p* < 0.001): it increased from day 0 to day 6, then plateaued. The rate also depended on the design: Z4-d3 scaffolds showed a 20%–161% increased proliferation rate compared to the other scaffold designs (*p* < 0.01). This is a typical pattern of cell growth of the MC3T3 cell line suggesting the end of cell proliferation, the initiation of cell differentiation and ECM formation, as well as lack of any prevalent cytotoxicity (Quarles et al., 1992; St-Pierre et al., 2005).

### 3.3 Cell viability

**FIGURE 5:**
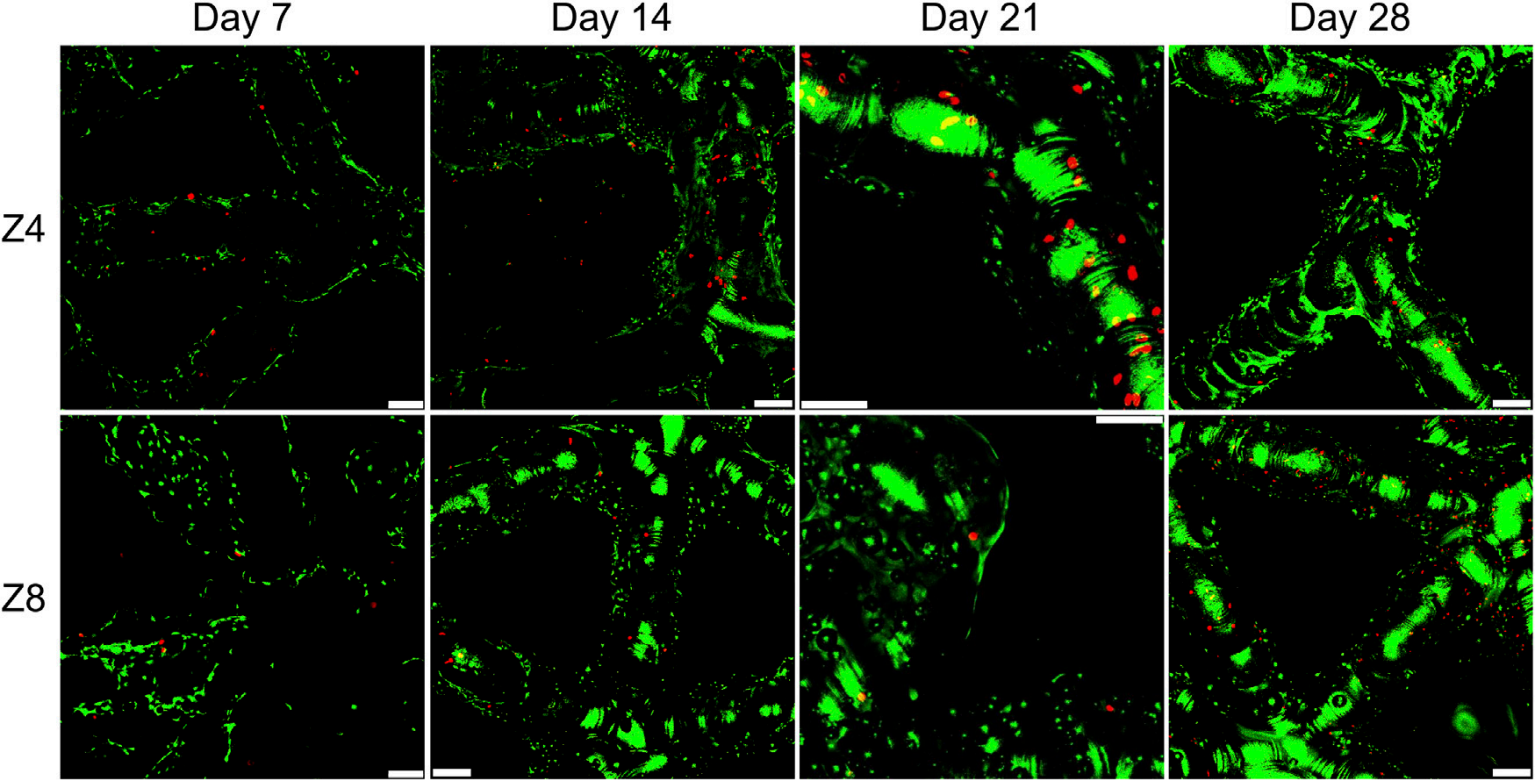
Fluorescence images showing live and dead staining on the top surface of scaffolds with different connectivity Z. All samples shown had strut density d = 3 struts/mm^3^. Green colour indicates live cells and red colour indicates dead cells. Images were taken using ×10 objective lens. Scale bars correspond to 100 μm in all images.

Representative fluorescent images of the live/dead assay at four time points were displayed in Figure 5 with similar characteristics seen at all designs. The pervasiveness of live cells over dead cells was apparent in all scaffolds, suggesting a high cell viability. Observation of the top and bottom surfaces of the scaffolds as well as their periphery demonstrated that cells successfully migrated inside the lattice structures of the scaffolds. Distinct cells spread along struts were seen at day 7, while scattered small green regions of cell clusters were seen after day 14 along struts and around the nodes. On day 21 and 28, larger green regions of more accumulated cell clusters were stained. These groups of cells were closer to each other covering larger areas of the scaffold’s network, indicative of new ECM and tissue formation.

### 3.4 Evaluation under SEM

**FIGURE 6:**
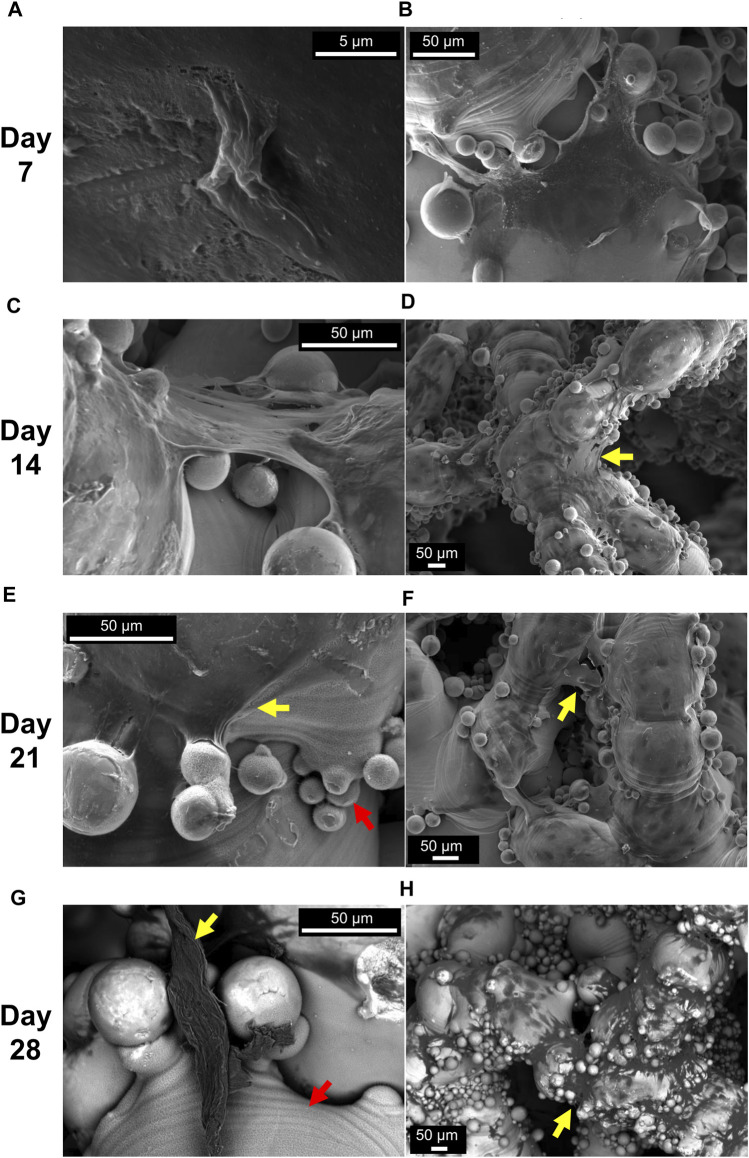
Representative SEM images at different days of cell culture (grouped by row). Isolated cells **(A)** and ECM formed at the nodes **(B)** were seen at day 7. Formed ECM bridging the corners of nodes were seen at day 14 **(C, D)** and day 21 **(E, F)**. Backscatter images show thick ECM parts anchored onto the coating porous film and ECM covering large areas of the struts at day 28 **(G, H)**. Yellow arrows indicate formed ECM and red arrows indicate the porous film due to NaOH treatment. Scaffold Z4-d3 is depicted in **(A, B, D)**; the Z4-d7 in **(F, G, H)**; the Z8-d3 in **(E)**; and the Z8-d7 in **(C)**

SEM images in Figure 6 highlight key aspects of cellular migration and proliferation inside porous scaffolds. Individual cells along struts (e.g., Figure 6A) and formed ECM at the corners (Figure 6B) were seen at day 7. In the early stages of cell culture (day 7 and 14), cells were spread and anchored around the semi-sintered powder particles (Figures 6B, C). Formed ECM bridging the corners of nodes was seen after day 14 (Figures 6D, F) with concave angles facilitating the phenomenon. Yet, the bridging of the corners was not as pronounced as expected, instead ECM was seen to wrap along struts’ length (Figure 6H). This can be attributed to the favourable “as-built” rough surface of the additively manufactured struts as well as the coating film due to the alkali treatment (notated with red arrows). SEM images, here, demonstrated that this thin micro-porous coating provided a cell-friendly substrate topography aiding the initial cell adhesion and later the anchoring and spreading of ECM inside the scaffolds (Figures 6E, G).

### 3.5 Collagen content

**FIGURE 7:**
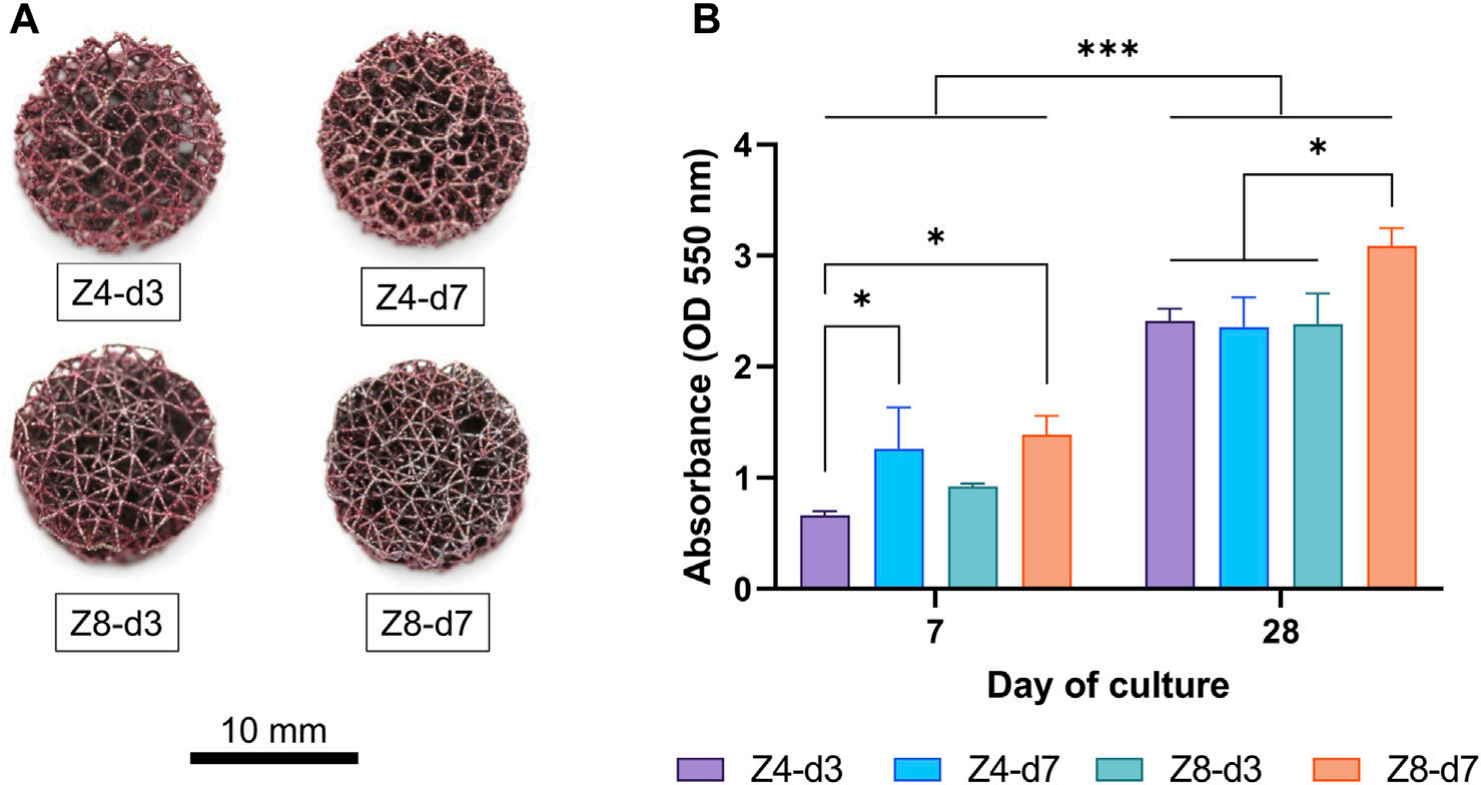
**(A)** Macroscopic images of scaffolds stained with picrosirius red at day 28. **(B)** Collagen content was quantified by measuring the fluorescence intensity of the bound dye. Each column corresponds to the mean value of *n* = 3. ANOVA results are provided in Supplementary Material (Supplementary Table S4).

Macroscopic images demonstrated that ECM rich in collagen content was formed deep inside all scaffolds and covered most of their surface area (Figure 7A). Measurements of the absorbance of dissolved dye are shown in Figure 7B. At day 7, both the higher strut density designs (Z8-d7 and Z4-d7) had more than double the collagen content compared to the low-low combination (Z4-d3, both *p* < 0.05). Collagen content increased by 87%–265% for all samples from day 7 to day 28 (*p* < 0.001) indicating the progressive growth and maturation of the ECM. By day 28, the densest scaffold (Z8-d7) had ∼30% more collagen accumulation that the other designs (*p* < 0.05).

### 3.6 Mineralised matrix labelling

**FIGURE 8:**
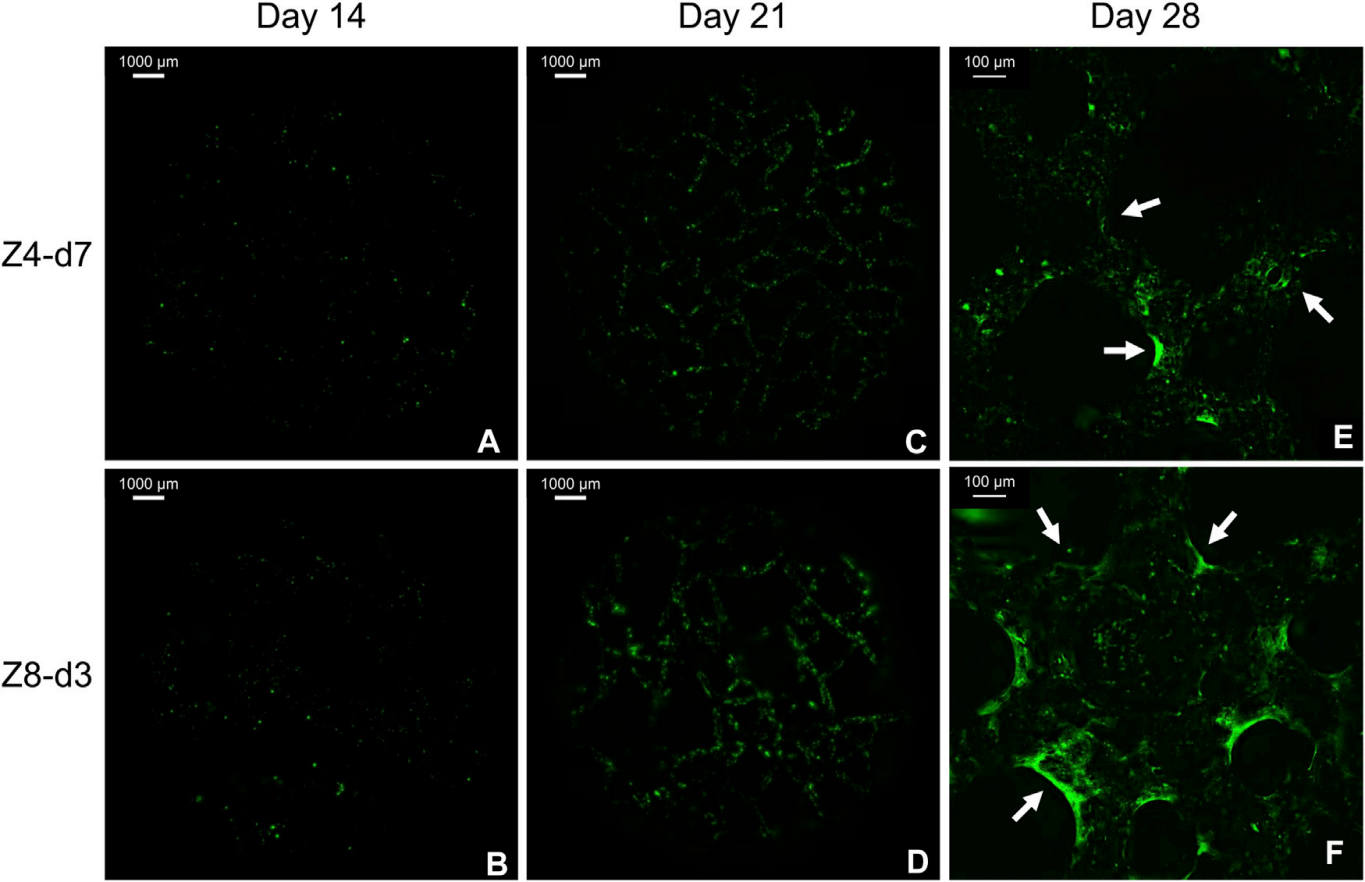
Fluorescent images from the top surface of scaffolds with similar porosity stained with Calcein. Macroscopic images from day 14 **(A, B)** and 21 **(C, D)** demonstrate the progress of mineralisation. Magnified images from day 28 **(E, F)** demonstrate the beneficial role of multiple corners at high connectivity scaffolds. Arrows indicate mineralised tissue bridging the corners.

As expected, no fluorescence was detected in any scaffold at day 7 as pre-osteoblasts were still on their proliferative phase. Minor green stained dots were observed at day 14 (Figures 8A, B) indicating the initiation of minerals deposition. At days 21 and 28 abundant green regions covered larger areas of the scaffolds’ surface (Figures 8C, D), indicating gradual mineralisation and bone tissue formation. At the end of the culture, extended mineralised zones were found at multiple regions (corners) around nodes mostly in high connectivity (Z8) scaffolds (Figure 8F). This differed from the low connectivity (Z4) scaffolds where mineralised ECM was observed on struts’ surface with limited bridging of the corners (Figure 8E).

### 3.7 Tissue maturation

**FIGURE 9:**
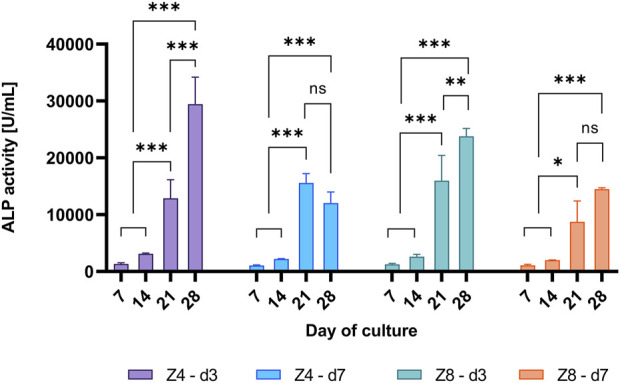
Measured ALP activity over different days of culture per scaffold design. Each column corresponds to the mean value of *n* = 3. ANOVA results are provided in Supplementary Material (Supplementary Table S5). Only differences within the sample design are shown.

Representative time-points (days 7, 14, 21 and 28) were chosen to compare the progress of pre-osteoblasts differentiation among designs. The ALP activity changed with day of culture (Figure 9). Enzyme’s activity only started to increase by day 14, where it increased by 314%–607% for all designs between days 14 and 21 in all designs (*p* < 0.05), indicating the initiation of cell differentiation. After 28 days, only the strut density d3 continued to express 49%–128% increased level of the enzyme’s activity (*p* < 0.05), while the activity was similar for the d7 designs.

**FIGURE 10:**
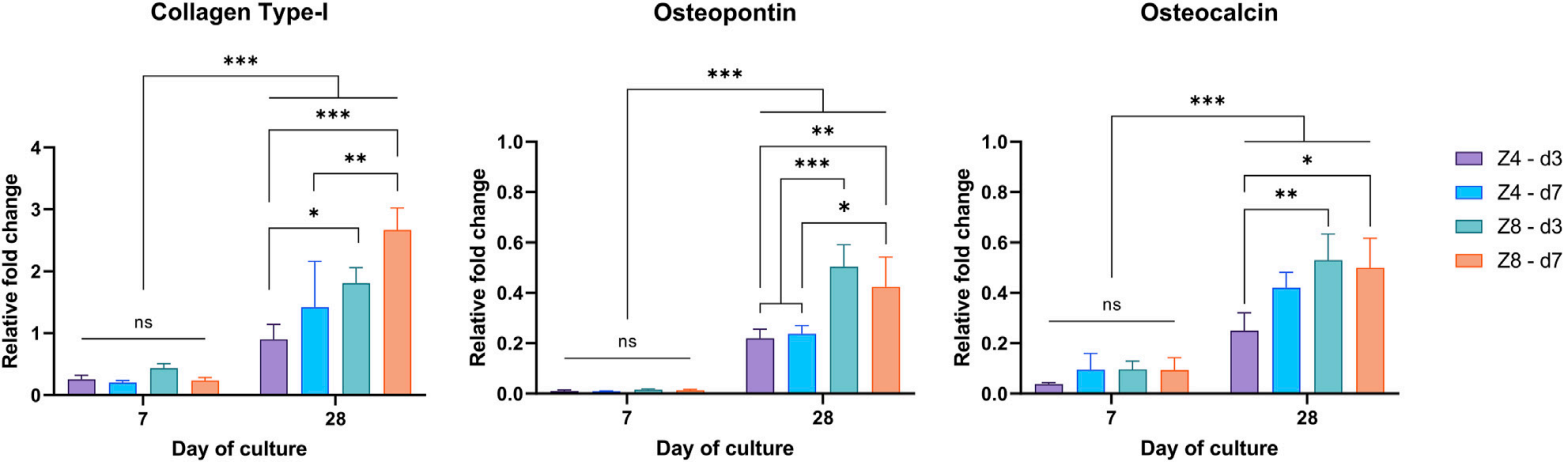
Measured expression levels of collagen type-I, osteopontin, and osteocalcin at days 7 and 28 through RT-PCR. Values normalised to *β*-actin. Each column corresponds to the mean value of *n* = 3. Results of the 3-way ANOVA are provided in Supplementary Material (Supplementary Tables S6–S8).

The expression levels of collagen type-I, osteopontin and osteocalcin at day 7 and day 28 are shown in Figure 10. Differences between designs were only apparent at day 28 (*p* < 0.05), agreeing with the ALP finding that cell differentiation had not measurably initiated by day 7. Thus, the levels of these (mainly late-stage) osteogenic biomarkers had more than doubled by day 28 compared to day 7 (*p* < 0.001). More importantly, though, different effect of each design parameter was observed depending on biomarker.

Connectivity and strut density alone were found to influence the expression of Collagen Type-I (*p* < 0.05). For fixed strut density, high connectivity (Z8) scaffolds had higher expression of collagen. While for fixed connectivity, scaffolds with higher strut density (d7) scaffolds exhibited relatively upregulated levels of collagen compared to low strut density (d3) scaffolds.

Only connectivity had affected the expression level of osteopontin (*p* < 0.05) with high connectivity (Z8) scaffolds exhibiting ∼2x more upregulated levels than low connectivity (Z4) scaffolds (*p* < 0.001). Lastly, connectivity (Z) also influenced the expression levels of Osteocalcin (*p* < 0.05), but it depended on the strut density. This can be perceived by the 112% increased levels of osteocalcin in the Z8 scaffolds compared to the Z4 scaffolds for the low strut density (d3).

## 4 Discussion

Nodal connectivity and strut density can be tailored to generate stochastic lattice structures that match the mechanical properties of trabecular bone. This study demonstrated how these design parameters can also regulate the micro-architecture and biological performance of cell-seeded scaffolds. Increasing strut density leads to denser structures with smaller pore size, while high connectivity, not only decreases porosity, but also results in tightly interconnected structures with increased surface area, numerous pores, and corners around nodes. Our findings suggest that increasing connectivity in stochastic designs is a novel technique to generate stiffness-matched scaffolds with a micro-architecture that favours osteoblastic differentiation.

### 4.1 Comparing the biological performance among designs

Additively manufactured titanium scaffolds with different geometrical characteristics were evaluated for their performance on cell adhesion, cell proliferation, cell differentiation and their ability to form bone-like tissue.

Equal number of cells was found in all scaffold designs 24 h after cell seeding, despite their different mean pore size which ranged from 680 to 1,040 μm. Our results differ from previous studies which evaluated cell adhesion (i.e., cell seeding efficiency) on periodic scaffolds of similar pore sizes (500–1,000 μm) (Van Bael et al., 2012; Ran et al., 2018). Specifically, in these studies, scaffolds with smaller pores demonstrated significantly higher cell attachment than scaffolds with larger pores, albeit scaffolds were made of the same material and pore geometry. Although cell adhesion on periodic scaffolds has been associated with the available surface area that cells can adhere to (Hsu et al., 2007), in our case scaffolds with different surface area demonstrated equivalent seeding efficiency and cell adhesion. This can be attributed the lower fluid permeability of stochastic lattices compared to periodic lattices (Stallard et al., 2023). And the difference in permeability has shown to originate from the increased tortuosity in stochastic micro-architectures (like the trabecular bone) compared to periodic ones (Prakoso et al., 2023). Ultimately, lower permeability and higher tortuosity result in lower velocity and impeded flow of the cell suspension during seeding which allows cells to attach on scaffold’s surface (Van Bael et al., 2012; Ali, 2019).

Regardless of the equal cell attachment on all scaffolds at day 1, the most porous scaffold (Z4-d3) exhibited the highest proliferation rate with cell number quadrupling the following days of culture, while less than threefold increase was seen in the rest of designs. These observations are in line with previous studies in periodic scaffolds with similar pore sizes (500–1,000 μm) (Van Bael et al., 2012; Ran et al., 2018), where larger pores have shown to induce higher cell proliferation. Nevertheless, colorimetric measurement of the bound Picrosirius Red indicated that the denser scaffold (Z8-d7) induced significant amount of collagen within expanded areas of newly formed ECM compared to the rest of designs. The contradiction between the prolonged cell proliferation in the most porous Z4-d3 compared to the enhanced ECM maturation in the densest Z8-d7 should be attributed to the combination of high connectivity, smaller mean pore size and larger surface area of the Z8-d7. High porosity results in uneven cell distribution inside scaffolds where cells attach to single struts distant to each other, while large pores further hinder cell migration to adjacent struts (Sobral et al., 2011; Van Bael et al., 2012). Small pores are known to facilitate cell migration and accelerate tissue formation due to smaller span gaps between struts (Frosch et al., 2002; Van Bael et al., 2012). The Z8-d7 was the design with the smallest pores, but also the one with the highest surface area (mainly due to numerous and more pronounced nodes). Given that all scaffolds demonstrated a wide range of pore size distribution with relatively large pores (>600 μm), we consider that the combination of high connectivity and high surface of nodes favoured cell-to-cell communication and cell differentiation over proliferation due to the accumulation of numerous cells over distinct locations (i.e., the nodes) inside the dense scaffolds. This suggests that the combination of scaffold porosity and topology (i.e., connectivity) drive differentiation independently to proliferation.

RT-qPCR analysis (Figure 9) further highlighted that both connectivity and strut density had a positive effect in osteoblast differentiation and ECM maturation. Increasing connectivity or strut density led to reduced porosity and mean pore size with simultaneous increase in surface area which favoured the expression of collagen type-I as suggested by previous *in vitro* studies (Chen et al., 2020).

This study employed pre-osteoblasts which are known to differentiate to mature osteo cells *a priori*. Thus, any differences in cellular behaviour found here should be attributed solely to scaffold designs. In fact, all scaffold designs supported osteoblastic differentiation and ECM formation and mineralisation as evinced by the positively expressed osteogenic biomarkers and the positive staining with calcein (Figure 8), respectively. Measurements of ALP activity, osteopontin, and osteocalcin indicate that both strut density and connectivity positively influenced cell differentiation. However, the expression of these proteins is known to be asynchronous, thus the time of their peak expression is key for comparing the progress of cell differentiation between designs (Weinreb et al., 1990).

During the pre-osteoblastic cycle, ALP is expressed when accumulated inorganic phosphate groups (Pi) are generated. Pi groups with calcium ions further deposit amorphous calcium phosphate or hydroxyapatite crystals within the secreted ECM (Vimalraj, 2020; Ansari et al., 2022). This is considered as an early phase marker of bone tissue formation, while at later phases ALP expression reaches a peak (plateau) value and finally decreases indicating the mineralisation of ECM (Choi et al., 1996; Beck et al., 1998; St-Pierre et al., 2005). This pattern was observed only in the d7 scaffolds (Figure 9), while the d3 scaffolds exhibited a continuously increasing activity of the enzyme suggesting either lag in cell differentiation or a state of continuous ECM mineralisation.

Osteopontin is a multifunctional protein which is secreted early by pre-osteoblasts to mediate cell adhesion and migration; however, its expression levels peak in more matured osteoblasts as it regulates collagen organisation and minerals deposition in ECM (SODEK et al., 1995; Carvalho et al., 2019). Osteocalcin, on the other hand, is principally secreted from mature osteoblasts as its main role is the maintenance of mineralised ECM as it binds to hydroxyapatite crystal (Hauschka and Wians, 1989; Chou et al., 2005). Prolonged culturing of MC3T3 pre-osteoblasts has shown that osteocalcin levels continuously increase over the days of culture (Choi et al., 1996). Higher expression in any of these two proteins due to higher strut density (d7) can again be attributed to the beneficial role of small pore size and high surface area (Chen et al., 2020). Still, connectivity appeared to have the most critical effect in the expression of both ostopontin and osteocalcin regardless of strut density.

Scaffolds Z4-d7 and Z8-d3 which exhibited similar porosity and pore sizes induced similar cell growth and expression levels of osteogenic biomarkers. This can be considered an expected outcome as the above morphological characteristics are known to be detrimental for cellular behaviour.

However, the parameters used to control scaffold design were connectivity and strut density. The existing results strongly support that, in all cases, increasing connectivity over fixed strut density favoured osteoblastic differentiation. This validated the initial hypothesis that high connectivity would benefit ECM organisation towards quicker maturation and mineralisation due to the presence of multiple cavities in the 3D structure as suggested by literature (Rumpler et al., 2008; Callens et al., 2020). High connectivity nodes were more pronounced than the low connectivity nodes, and as a result, they provided more available surface area at the junction of the struts for cells to accumulate and form ECM. After 2 weeks, the Z8 scaffolds demonstrated distinguishable bridging of multiple corners around the nodes, compared to the Z4 scaffolds where ECM was mainly formed around the struts.

Out of all the designs, the dense Z8-d7 combining the highest tested values of strut density and connectivity demonstrated the most accelerated osteoblastic and ECM maturation. This scaffold was the only with plateau in the ALP expression and highly expressed osteopontin and osteocalcin at the end of culture (day 28). At the same time, it led to the largest ECM formation (shown through Picrosirius Red staining) and the most matured ECM (highest expression of collagen type-I). These findings suggest that the ultimate goal in stochastic scaffold design should focus on maximising the values of both strut density and connectivity while assuring that structure maintains a stiffness in the range found in natural trabecular bone.

### 4.2 A design framework for stochastic lattices

*In vivo* animal trials (Pobloth et al., 2018; Reznikov et al., 2019) suggest that bone in-growth and the quality of regenerated tissue (lamellar *versus* woven bone) highly improves when scaffold stiffness matches the stiffness of natural bone. This is attributed to the positive bone remodelling of host bone due to the generation of physiological strains upon implantation. Taking this observation into account, scaffold designs can be designed and fabricated using our stochastic model to match the properties of natural bone (Kechagias et al., 2022). Strut density was selected to lay in the range of trabeculae density (1/mm^3^) found in natural bone (Odgaard and Gundersen, 1993). Nodal connectivity in natural bone ranges between 3 and 5 (Reznikov et al., 2016), thus the majority of stochastic lattices found in literature are designed with low connectivity (Reznikov et al., 2019; Wang et al., 2021; Jiao et al., 2023). Previous experimental data (Ahmadi et al., 2018; Zhao et al., 2018; Kechagias et al., 2022) suggest that increasing connectivity can lead to significant improvement of static and fatigue strength of lattices due to more tightly interconnected struts, while the current study further demonstrated that high connectivity positively influences cellular behaviour.

Critical sized implants, however, are subject to gradient loads thus they should combine regions of high and low porosities. Lattice designs with locally graded porosity to match the stiffness of bone (Wieding et al., 2014; Ghouse et al., 2019) and induce high strains to the host bone (Shum et al., 2022) have been previously proposed to mechanically stimulate osseointegration. In most cases, periodic lattices with varied strut thickness or unit cell type/size were used. However, these approaches require a variety of known laser parameters to fabricate several strut thicknesses or introduce issues of unit cells mismatch (Zhang et al., 2018; Kechagias et al., 2022). Varying strut thickness remains the gold standard for generating graded structures, however rigorous mechanical testing of each set of laser parameters is required to accurately control strut and structure properties (Ahmadi et al., 2017; Hossain et al., 2021b). Stochastic lattice structures with locally varied properties can be designed using different combinations of strut density and connectivity using constant strut thickness, while offering high isotropy throughout the structure (struts are found in all directions compared to periodic lattices) (Kechagias et al., 2022).

High load-bearing lattices are typically designed by decreasing pore size and increasing strut density. Extreme strut densities accompanied with low connectivity will eventually lead to very small pore sizes that will hinder cell migration and vascularisation *in vivo* (Taniguchi et al., 2016; Ran et al., 2018; Kelly et al., 2021), while the design of large-scale fully porous implants requires generally considerably higher computational times (Burge et al., 2023). Design of stiffness-matched lattices with increased connectivity and adjusted strut density constitutes a solid alternative for combining high mechanical endurance while allowing room for deep bone in-growth and vascularisation.

### 4.3 Limitations and considerations for future work

Our image-based analysis has several limitations. Firstly, our observations emphasized on the distribution of cells and ECM mainly on the top layers and the periphery of scaffolds. Cutting the specimens and observing the extent of cell penetration and tissue formation towards the middle of the scaffolds would provide valuable insights for the development of large-scale porous implants. However, this would require fixation in epoxy resin to avoid damage of the cellular structure and the scaffold material due to high-speed cutting. Secondly, monitoring cell proliferation and ECM expansion along struts and around nodes at higher magnification would better illustrate how connectivity and strut dimensions guide tissue formation. Such study, however, would require numerous timepoints.

Surface roughness has shown to benefit cell adhesion and proliferation when it is comparable to cell size (<10 μm) (Bigerelle et al., 2002). Strut roughness in the tested scaffolds was mainly above this limit, with the semi-sintered particles acting as surface discontinuities which led cells to cluster and secrete ECM around them. Although, surface polishing of titanium scaffolds (Maleki et al., 2021) has not shown to induce differences in osteoblastic activity *in vitro* (Wysocki et al., 2019; Rovetta et al., 2023), removal of the protruding semi-sintered particles can induce several advantages for future clinical application. First, it can further increase surface wettability and hydrophilicity (Rovetta et al., 2023), second it can increase the fatigue life of lattices (Van Hooreweder et al., 2017), and third it can minimise the chances of bone resorption that can happen *in vivo* due to floating metallic debris (Vasconcelos et al., 2016). Moreover, coating the titanium surface would further enhance the bioactivity of scaffolds. Techniques such as plasma spraying, electrochemical deposition and sol-gel have previously been used to coat solid titanium implants with hydroxyapatite or calcium phosphate compounds (Drevet et al., 2023) and could potentially applied to lattice structures too.

This *in vitro* study was a cell-only model which emphasized on the influence of two key design parameters on cellular behaviour. We aimed to make this study as detailed as possible by studying four novel designs at a span of 4 weeks. A variety of different techniques collectively supported the raised arguments through complementary optical and quantitative means. However, cell-only models are limited in that they do not consider cell migration from native tissue, the influence of physiological loading, or vascularisation. A next step could be to consider how cells migrate from bone tissue into lattices and the effect of physiological loading using an *ex vivo* model (Kohli et al., 2023). Hypoxic culturing conditions should also be implemented in future lab-based works to better simulate the environment of natural bone–also known to regulate osteo-angiogenesis (Sheehy et al., 2012; Usategui-Martín et al., 2022). Ultimately, the interplay between bone formation and vascularisation needs to be examined with an *in vivo* model.

More *in vivo* data are needed to outline how scaffold morphology and surface properties affect the bone-implant interface and how the quality of tissue formed at this interface is changing over long periods of healing (Shah et al., 2019; Stich et al., 2022). Additionally, while the use of cell-seeded scaffolds has previously shown promising results for *in vivo* bone regeneration in biodegradable scaffolds (Shin et al., 2004; Mauney et al., 2005), there has been a lack of recent investigation of pre-seeded titanium implants.

Finally, our results suggest that all scaffold designs can support bone-like tissue formation, albeit with different *in vitro* maturation time. Yet, the porous structure and properties of trabecular bone varies depending on the anatomical site, age, sex, activity level and health condition (Ghouse et al., 2019). Consequently, future research should also focus on the production of anatomical site or patient-specific implants by investigating how topologically different stochastic scaffolds perform depending on the nature of the host bone.

## 5 Conclusion

• Nodal connectivity and strut density both positively influence cell differentiation, ECM formation and maturation.
• Stochastic scaffolds enabled equivalent cell seeding among designs with diverge surface areas and porosities, contrary to what is usually observed in periodic scaffolds.
• High porosity (>90%) and large pore size (>1000 μm) promote increased cell proliferation while suppressing cell differentiation. This suggests that the qualitative tissue formation in stochastic scaffolds is not correlated with cell proliferation but rather with the scaffold’s internal geometry.
• High connectivity had the most pronounced effect on osteoblastic differentiation due the increased surface area and number of connections around the nodes.
• Increasing connectivity and adjusting strut density to match the properties of natural bone is a novel technique offering high mechanical endurance, high porosity, and favourable micro-architecture for rapid ossification.
• The presented design approach provides high design freedom and enables the integration of stochastic lattices into large-scale implants with bespoken shape and mechanical properties.

## Data Availability

The original contributions presented in the study are included in the article/Supplementary Material, further inquiries can be directed to the corresponding authors.

## References

[B1] Ahmadi S. M. Hedayati R. Ashok Kumar Jain R. K. Li Y. Leeflang S. Zadpoor A. A. (2017). Effects of laser processing parameters on the mechanical properties, topology, and microstructure of additively manufactured porous metallic biomaterials: a vector-based approach. Mater Des. 134, 234–243. 10.1016/j.matdes.2017.08.046

[B2] Ahmadi S. M. Hedayati R. Li Y. Lietaert K. Tümer N. Fatemi A. (2018). Fatigue performance of additively manufactured meta-biomaterials: the effects of topology and material type. Acta Biomater. 65, 292–304. 10.1016/j.actbio.2017.11.014 29127065

[B3] Albrektsson T. Johansson C. (2001). Osteoinduction, osteoconduction and osseointegration. Eur. Spine J. 10, S96–S101. 10.1007/s005860100282 11716023 PMC3611551

[B4] Ali D. (2019). Effect of scaffold architecture on cell seeding efficiency: a discrete phase model CFD analysis. Comput. Biol. Med. 109, 62–69. 10.1016/j.compbiomed.2019.04.025 31035072

[B5] Ansari S. Ito K. Hofmann S. (2022). Alkaline phosphatase activity of serum affects osteogenic differentiation cultures. ACS Omega 7 (15), 12724–12733. 10.1021/acsomega.1c07225 35474849 PMC9026015

[B6] Ashby M. F. (2006). The properties of foams and lattices. Philosophical Trans. R. Soc. A Math. Phys. Eng. Sci. 364, 15–30. 10.1098/rsta.2005.1678 18272451

[B7] Beck G. R. Sullivan E. C. Moran E. Zerler B. (1998). Relationship between alkaline phosphatase levels, osteopontin expression, and mineralization in differentiating mc3t3-E1 osteoblasts. J. Cell. Biochem. 68, 269–280. 10.1002/(sici)1097-4644(19980201)68:2<269::aid-jcb13>3.0.co;2-a 9443082

[B8] Bidan C. M. Kommareddy K. P. Rumpler M. Kollmannsberger P. Fratzl P. Dunlop J. W. C. (2013). Geometry as a factor for tissue growth: towards shape optimization of tissue engineering scaffolds. Adv. Healthc. Mater 2 (1), 186–194. 10.1002/adhm.201200159 23184876

[B9] Bigerelle M. Anselme K. Noël B. Ruderman I. Hardouin P. Iost A. (2002). Improvement in the morphology of Ti-based surfaces: a new process to increase *in vitro* human osteoblast response. Biomaterials 23 (7), 1563–1577. 10.1016/s0142-9612(01)00271-x 11922462

[B10] Buenzli P. R. Lanaro M. Wong C. S. McLaughlin M. P. Allenby M. C. Woodruff M. A. (2020). Cell proliferation and migration explain pore bridging dynamics in 3D printed scaffolds of different pore size. Acta Biomater. 114, 285–295. 10.1016/j.actbio.2020.07.010 32673750

[B11] Burge T. A. Munford M. J. Kechagias S. Jeffers J. R. T. Myant C. W. (2023). Automating the customization of stiffness-matched knee implants using machine learning techniques. Int. J. Adv. Manuf. Technol. 126, 3725–3737. 10.1007/s00170-023-11357-6

[B12] Callens S. J. P. Uyttendaele R. J. C. Fratila-Apachitei L. E. Zadpoor A. A. (2020). Substrate curvature as a cue to guide spatiotemporal cell and tissue organization. Biomaterials 232, 119739. 10.1016/j.biomaterials.2019.119739 31911284

[B13] Carvalho M. S. Cabral J. M. S. da Silva C. L. Vashishth D. (2019). Synergistic effect of extracellularly supplemented osteopontin and osteocalcin on stem cell proliferation, osteogenic differentiation, and angiogenic properties. J. Cell. Biochem. 120 (4), 6555–6569. 10.1002/jcb.27948 30362184

[B14] Chen H. Han Q. Wang C. Liu Y. Chen B. Wang J. (2020a). Porous scaffold design for additive manufacturing in orthopedics: a review. Front. Bioeng. Biotechnol. 8 (June), 609–620. 10.3389/fbioe.2020.00609 32626698 PMC7311579

[B15] Chen Z. Yan X. Yin S. Liu L. Liu X. Zhao G. (2020b). Influence of the pore size and porosity of selective laser melted Ti6Al4V ELI porous scaffold on cell proliferation, osteogenesis and bone ingrowth. Mater. Sci. Eng. C 106, 110289. 10.1016/j.msec.2019.110289 31753386

[B16] Choi J. Y. Lee B. H. Song K. B. Park R. W. Kim I. S. Sohn K. Y. (1996). Expression patterns of bone-related proteins during osteoblastic differentiation in MC3T3-E1 cells. J. Cell. Biochem. 61 (4), 609–618. 10.1002/(sici)1097-4644(19960616)61:4<609::aid-jcb15>3.0.co;2-a 8806085

[B17] Chou Y. F. Dunn J. C. Y. Wu B. M. (2005). *In vitro* response of MC3T3‐E1 preosteoblasts within three‐dimensional apatite‐coated PLGA scaffolds. J. Biomed. Mater Res. 75B (1), 81–90. 10.1002/jbm.b.30261 16001421

[B18] Deering J. Grandfield K. (2021). Current interpretations on the *in vivo* response of bone to additively manufactured metallic porous scaffolds: a review. Biomaterials Biosyst. 2, 100013. 10.1016/j.bbiosy.2021.100013 PMC9934422 36824658

[B19] Drevet R. Fauré J. Benhayoune H. (2023). Bioactive calcium phosphate coatings for bone implant applications: a review. Coatings 13, 1091. 10.3390/coatings13061091

[B20] Emmelmann C. Scheinemann P. Munsch M. Seyda V. (2011). Laser additive manufacturing of modified implant surfaces with osseointegrative characteristics. Phys. Procedia 12 (PART 1), 375–384. 10.1016/j.phpro.2011.03.048

[B21] Frosch K. H. Barvencik F. Lohmann C. H. Viereck V. Siggelkow H. Breme J. (2002). Migration, matrix production and lamellar bone formation of human osteoblast-like cells in porous titanium implants. Cells Tissues Organs 170, 214–227. 10.1159/000047925 11919409

[B22] Ghouse S. Babu S. Van Arkel R. J. Nai K. Hooper P. A. Jeffers J. R. T. (2017). The influence of laser parameters and scanning strategies on the mechanical properties of a stochastic porous material. Mater Des. 131, 498–508. 10.1016/j.matdes.2017.06.041

[B23] Ghouse S. Reznikov N. Boughton O. R. Babu S. Ng K. C. G. Blunn G. (2019). The design and *in vivo* testing of a locally stiffness-matched porous scaffold. Appl. Mater Today 15, 377–388. 10.1016/j.apmt.2019.02.017 31281871 PMC6609455

[B24] Hadjidakis D. J. Androulakis II (2006). Bone remodeling. Ann. N. Y. Acad. Sci. 1092, 385–396. 10.1196/annals.1365.035 17308163

[B25] Hale L. V. Ma Y. F. Santerre R. F. (2000). Semi-quantitative fluorescence analysis of calcein binding as a measurement of *in vitro* mineralization. Calcif. Tissue Int. 67, 80–84. 10.1007/s00223001101 10908418

[B26] Hauschka P. V. Wians F. H. Jr. (1989). Osteocalcin-hydroxyapatite interaction in the extracellular organic matrix of bone. Anat. Rec. 224 (2), 180–188. 10.1002/ar.1092240208 2549810

[B27] He X. Huang Z. Liu W. Liu Y. Qian H. Lei T. (2021). Electrospun polycaprolactone/hydroxyapatite/ZnO films as potential biomaterials for application in bone-tendon interface repair. Colloids Surf. B Biointerfaces 204, 111825. 10.1016/j.colsurfb.2021.111825 33984615

[B28] Hildebrand T. Ruegsegger P. (1997). A new method for the model-independent assessment of thickness in three-dimensional images. J. Microsc. 185 (1), 67–75. 10.1046/j.1365-2818.1997.1340694.x

[B29] Hossain U. Ghouse S. Nai K. Jeffers J. R. (2021a). Controlling and testing anisotropy in additively manufactured stochastic structures. Addit. Manuf. 39, 101849. 10.1016/j.addma.2021.101849 PMC8448581 34603974

[B30] Hossain U. Ghouse S. Nai K. Jeffers J. R. T. (2021b). Mechanical and morphological properties of additively manufactured SS316L and Ti6Al4V micro-struts as a function of build angle. Addit. Manuf. 46, 102050. 10.1016/j.addma.2021.102050 PMC8448581 34603974

[B31] Hsu S. Yen H. J. Tseng C. S. Cheng C. S. Tsai C. L. (2007). Evaluation of the growth of chondrocytes and osteoblasts seeded into precision scaffolds fabricated by fused deposition manufacturing. J. Biomed. Mater Res. B Appl. Biomater. 80B (2), 519–527. 10.1002/jbm.b.30626 16862556

[B32] International Organization for Standardization (2011). ISO 13314:2011 Mechanical testing of metals – ductility testing – compression test for porous and cellular metals. International Standard.

[B33] Jiao J. Hong Q. Zhang D. Wang M. Tang H. Yang J. (2023). Influence of porosity on osteogenesis, bone growth and osteointegration in trabecular tantalum scaffolds fabricated by additive manufacturing. Front. Bioeng. Biotechnol. 11, 1117954. 10.3389/fbioe.2023.1117954 36777251 PMC9911888

[B34] Joshi M. G. Advani S. G. Miller F. Santare M. H. (2000). Analysis of a femoral hip prosthesis designed to reduce stress shielding. J. Biomech. 33 (12), 1655–1662. 10.1016/s0021-9290(00)00110-x 11006390

[B35] Karageorgiou V. Kaplan D. (2005). Porosity of 3D biomaterial scaffolds and osteogenesis. Biomaterials 26 (27), 5474–5491. 10.1016/j.biomaterials.2005.02.002 15860204

[B36] Kechagias S. Oosterbeek R. N. Munford M. J. Ghouse S. Jeffers J. R. T. (2022). Controlling the mechanical behaviour of stochastic lattice structures: the key role of nodal connectivity. Addit. Manuf. 54, 102730. 10.1016/j.addma.2022.102730

[B37] Kelly C. N. Wang T. Crowley J. Wills D. Pelletier M. H. Westrick E. R. (2021). High-strength, porous additively manufactured implants with optimized mechanical osseointegration. Biomaterials 279, 121206. 10.1016/j.biomaterials.2021.121206 34715639

[B38] Kersh M. E. Zysset P. K. Pahr D. H. Wolfram U. Larsson D. Pandy M. G. (2013). Measurement of structural anisotropy in femoral trabecular bone using clinical-resolution CT images. J. Biomech. 46 (15), 2659–2666. 10.1016/j.jbiomech.2013.07.047 24007613

[B39] Kim C. Kendall M. R. Miller M. A. Long C. L. Larson P. R. Humphrey M. B. (2013). Comparison of titanium soaked in 5 M NaOH or 5 M KOH solutions. Mater. Sci. Eng. C 33 (1), 327–339. 10.1016/j.msec.2012.08.047 PMC3613859 23565038

[B40] Knychala J. Bouropoulos N. Catt C. J. Katsamenis O. L. Please C. P. Sengers B. G. (2013). Pore geometry regulates early stage human bone marrow cell tissue formation and organisation. Ann. Biomed. Eng. 41 (5), 917–930. 10.1007/s10439-013-0748-z 23404072

[B41] Kohli N. Theodoridis K. Hall T. A. G. Sanz-Pena I. Gaboriau D. C. A. van Arkel R. J. (2023). Bioreactor analyses of tissue ingrowth, ongrowth and remodelling around implants: an alternative to live animal testing. Front. Bioeng. Biotechnol. 11, 1054391. 10.3389/fbioe.2023.1054391 36890911 PMC9986429

[B42] Liang H. Chao L. Xie D. Yang Y. Shi J. Zhang Y. (2022). Trabecular-like Ti–6Al–4V scaffold for bone repair: a diversified mechanical stimulation environment for bone regeneration. Compos B Eng. 241, 110057. 10.1016/j.compositesb.2022.110057

[B43] Liang H. Yang Y. Xie D. Li L. Mao N. Wang C. (2019). Trabecular-like Ti-6Al-4V scaffolds for orthopedic: fabrication by selective laser melting and *in vitro* biocompatibility. J. Mater Sci. Technol. 35 (7), 1284–1297. 10.1016/j.jmst.2019.01.012

[B44] Liang H. Yu F. (2018). Quercetin promotes MC3T3-E1 cell growth via PI3K/AKt signaling pathway. Trop. J. Pharm. Res. 17 (12), 2371–2374. 10.4314/tjpr.v17i12.8

[B45] Maleki E. Bagherifard S. Bandini M. Guagliano M. (2021). Surface post-treatments for metal additive manufacturing: progress, challenges, and opportunities. Addit. Manuf. 37 (July), 101619. 10.1016/j.addma.2020.101619

[B46] Man K. Brunet M. Y. Louth S. Robinson T. E. Fernandez-Rhodes M. Williams S. (2021). Development of a bone-mimetic 3D printed Ti6Al4V scaffold to enhance osteoblast-derived extracellular vesicles’ therapeutic efficacy for bone regeneration. Front. Bioeng. Biotechnol. 9, 757220. 10.3389/fbioe.2021.757220 34765595 PMC8576375

[B47] Markhoff J. Wieding J. Weissmann V. Pasold J. Jonitz-Heincke A. Bader R. (2015). Influence of different three-dimensional open porous titanium scaffold designs on human osteoblasts behavior in static and dynamic cell investigations. Materials 8 (8), 5490–5507. 10.3390/ma8085259 28793519 PMC5455497

[B48] Mauney J. R. Jaquiéry C. Volloch V. Heberer M. Martin I. Kaplan D. L. (2005). *In vitro* and *in vivo* evaluation of differentially demineralized cancellous bone scaffolds combined with human bone marrow stromal cells for tissue engineering. Biomaterials 26 (16), 3173–3185. 10.1016/j.biomaterials.2004.08.020 15603812

[B49] Morgan E. F. Bayraktar H. H. Keaveny T. M. (2003). Trabecular bone modulus–density relationships depend on anatomic site. J. Biomech. 36 (7), 897–904. 10.1016/s0021-9290(03)00071-x 12757797

[B50] Nune K. C. Misra R. D. K. Li S. J. Hao Y. L. Yang R. (2016). Cellular response of osteoblasts to low modulus Ti-24Nb-4Zr-8Sn alloy mesh structure. J. Biomed. Mater Res. Part A 105, 859–870. 10.1002/jbm.a.35963 27885781

[B51] Odgaard A. Gundersen H. J. G. (1993). Quantification of connectivity in cancellous bone, with special emphasis on 3-D reconstructions. Bone 14, 173–182. 10.1016/8756-3282(93)90245-6 8334036

[B52] Pobloth A. M. Checa S. Razi H. Petersen A. Weaver J. C. Schmidt-Bleek K. (2018). Mechanobiologically optimized 3D titanium-mesh scaffolds enhance bone regeneration in critical segmental defects in sheep. Sci. Transl. Med. 10, eaam8828. 10.1126/scitranslmed.aam8828 29321260

[B53] Prakoso A. T. Basri H. Adanta D. Yani I. Ammarullah M. I. Akbar I. (2023). The effect of tortuosity on permeability of porous scaffold. Biomedicines 11 (2), 427. 10.3390/biomedicines11020427 36830961 PMC9953537

[B54] Quarles L. D. Yohay D. A. Lever L. W. Caton R. Wenstrup R. J. (1992). Distinct proliferative and differentiated stages of murine MC3T3‐E1 cells in culture: an *in vitro* model of osteoblast development. J. Bone Mineral Res. 7 (6), 683–692. 10.1002/jbmr.5650070613 1414487

[B55] Ran Q. Yang W. Hu Y. Shen X. Yu Y. Xiang Y. (2018). Osteogenesis of 3D printed porous Ti6Al4V implants with different pore sizes. J. Mech. Behav. Biomed. Mater 84, 1–11. 10.1016/j.jmbbm.2018.04.010 29709846

[B56] Reznikov N. Boughton O. R. Ghouse S. Weston A. E. Collinson L. Blunn G. W. (2019). Individual response variations in scaffold-guided bone regeneration are determined by independent strain- and injury-induced mechanisms. Biomaterials 194, 183–194. 10.1016/j.biomaterials.2018.11.026 30611115 PMC6345626

[B57] Reznikov N. Chase H. Ben Zvi Y. Tarle V. Singer M. Brumfeld V. (2016). Inter-trabecular angle: a parameter of trabecular bone architecture in the human proximal femur that reveals underlying topological motifs. Acta Biomater. 44, 65–72. 10.1016/j.actbio.2016.08.040 27554017

[B58] Rovetta R. Ginestra P. Ferraro R. M. Zohar-Hauber K. Giliani S. Ceretti E. (2023). Building orientation and post processing of Ti6Al4V produced by laser powder bed fusion process. J. Manuf. Mater. Process. 7 (1), 43. 10.3390/jmmp7010043

[B59] Rumpler M. Woesz A. Dunlop J. W. C. Van Dongen J. T. Fratzl P. (2008). The effect of geometry on three-dimensional tissue growth. J. R. Soc. Interface 5 (27), 1173–1180. 10.1098/rsif.2008.0064 18348957 PMC2495039

[B60] Shah F. A. Thomsen P. Palmquist A. (2019). Osseointegration and current interpretations of the bone-implant interface. Acta Biomater. 84, 1–15. 10.1016/j.actbio.2018.11.018 30445157

[B61] Sheehy E. J. Buckley C. T. Kelly D. J. (2012). Oxygen tension regulates the osteogenic, chondrogenic and endochondral phenotype of bone marrow derived mesenchymal stem cells. Biochem. Biophys. Res. Commun. 417 (1), 305–310. 10.1016/j.bbrc.2011.11.105 22155244

[B62] Shin M. Yoshimoto H. Vacanti J. P. (2004). *In vivo* bone tissue engineering using mesenchymal stem cells on a novel electrospun nanofibrous scaffold. Tissue Eng. 10 (1–2), 33–41. 10.1089/107632704322791673 15009928

[B63] Shum J. M. Gadomski B. C. Tredinnick S. J. Fok W. Fernandez J. Nelson B. (2022). Enhanced bone formation in locally-optimised, low-stiffness additive manufactured titanium implants: an *in silico* and *in vivo* tibial advancement study. Acta Biomater. 156, 202–213. 10.1016/j.actbio.2022.04.006 35413478

[B64] Sobral J. M. Caridade S. G. Sousa R. A. Mano J. F. Reis R. L. (2011). Three-dimensional plotted scaffolds with controlled pore size gradients: effect of scaffold geometry on mechanical performance and cell seeding efficiency. Acta Biomater. 7 (3), 1009–1018. 10.1016/j.actbio.2010.11.003 21056125

[B65] Sodek J. Chen J. Nagata T. Kasugai S. Todescan R. Li I. W. S. (1995). Regulation of osteopontin expression in osteoblasts. Ann. N. Y. Acad. Sci. 760 (1), 223–241. 10.1111/j.1749-6632.1995.tb44633.x 7785896

[B66] Stallard S. Jiang H. Chen Y. Bergman T. L. Li X. (2023). Exploring the design space of the effective thermal conductivity, permeability, and stiffness of high-porosity foams. Mater Des. 231, 112027. 10.1016/j.matdes.2023.112027

[B67] Stich T. Alagboso F. Křenek T. Kovářík T. Alt V. Docheva D. (2022). Implant-bone-interface: reviewing the impact of titanium surface modifications on osteogenic processes *in vitro* and *in vivo* . Bioeng. Transl. Med. 7, e10239. 10.1002/btm2.10239 35079626 PMC8780039

[B68] St-Pierre J. P. Gauthier M. Lefebvre L. P. Tabrizian M. (2005). Three-dimensional growth of differentiating MC3T3-E1 pre-osteoblasts on porous titanium scaffolds. Biomaterials 26 (35), 7319–7328. 10.1016/j.biomaterials.2005.05.046 16000220

[B69] Taniguchi N. Fujibayashi S. Takemoto M. Sasaki K. Otsuki B. Nakamura T. (2016). Effect of pore size on bone ingrowth into porous titanium implants fabricated by additive manufacturing: an *in vivo* experiment. Mater. Sci. Eng. C 59, 690–701. 10.1016/j.msec.2015.10.069 26652423

[B70] Tekogu C. Gibson L. J. Pardoen T. Onck P. R. (2011). Size effects in foams: experiments and modeling. Prog. Mater Sci. 56 (2), 109–138. 10.1016/j.pmatsci.2010.06.001

[B71] Torres-Sanchez C. Borgman J. M. Sargeant B. Bell H. Alabort E. Lindsay C. (2022). Comparison of selective laser melted commercially pure titanium sheet-based triply periodic minimal surfaces and trabecular-like strut-based scaffolds for tissue engineering. Adv. Eng. Mater 24 (1). 10.1002/adem.202270002

[B72] Usategui-Martín R. Rigual R. Ruiz-Mambrilla M. Fernández-Gómez J. M. Dueñas A. Pérez-Castrillón J. L. (2022). Molecular mechanisms involved in hypoxia-induced alterations in bone remodeling. Int. J. Mol. Sci. 23, 3233. 10.3390/ijms23063233 35328654 PMC8953213

[B73] Van Bael S. Chai Y. C. Truscello S. Moesen M. Kerckhofs G. Van Oosterwyck H. (2012). The effect of pore geometry on the *in vitro* biological behavior of human periosteum-derived cells seeded on selective laser-melted Ti6Al4V bone scaffolds. Acta Biomater. 8 (7), 2824–2834. 10.1016/j.actbio.2012.04.001 22487930

[B74] Van Hooreweder B. Apers Y. Lietaert K. Kruth J. P. (2017). Improving the fatigue performance of porous metallic biomaterials produced by Selective Laser Melting. Acta Biomater. 47, 193–202. 10.1016/j.actbio.2016.10.005 27717912

[B75] Vasconcelos D. M. Santos S. G. Lamghari M. Barbosa M. A. (2016). The two faces of metal ions: from implants rejection to tissue repair/regeneration. Biomaterials 84, 262–275. 10.1016/j.biomaterials.2016.01.046 26851391

[B76] Vimalraj S. (2020). Alkaline phosphatase: structure, expression and its function in bone mineralization. Gene 754, 144855. 10.1016/j.gene.2020.144855 32522695

[B77] Vogel V. Sheetz M. (2006). Local force and geometry sensing regulate cell functions. Nat. Rev. Mol. Cell. Biol. 7, 265–275. 10.1038/nrm1890 16607289

[B78] Wang C. Xu D. Li S. Yi C. Zhang X. He Y. (2020). Effect of pore size on the physicochemical properties and osteogenesis of Ti6Al4V porous scaffolds with bionic structure. ACS Omega 5 (44), 28684–28692. 10.1021/acsomega.0c03824 33195921 PMC7658928

[B79] Wang C. Xu D. Lin L. Li S. Hou W. He Y. (2021). Large-pore-size Ti6Al4V scaffolds with different pore structures for vascularized bone regeneration. Mater. Sci. Eng. C 131, 112499. 10.1016/j.msec.2021.112499 34857285

[B80] Wang Z. Wang C. Li C. Qin Y. Zhong L. Chen B. (2017). Analysis of factors influencing bone ingrowth into three-dimensional printed porous metal scaffolds: a review. J. Alloys Compd. 717, 271–285. 10.1016/j.jallcom.2017.05.079

[B81] Weinreb M. Shinar D. Rodan G. A. (1990). Different pattern of alkaline phosphatase, osteopontin, and osteocalcin expression in developing rat bone visualized by *in situ* hybridization. J. Bone Mineral Res. 5 (8), 831–842. 10.1002/jbmr.5650050806 2239367

[B82] Wieding J. Wolf A. Bader R. (2014). Numerical optimization of open-porous bone scaffold structures to match the elastic properties of human cortical bone. J. Mech. Behav. Biomed. Mater 37, 56–68. 10.1016/j.jmbbm.2014.05.002 24942627

[B83] Wilson C. J. Clegg R. E. Leavesley D. I. Pearcy M. J. (2005). Mediation of biomaterial–cell interactions by adsorbed proteins: a review. Tissue Eng. 11 (1–2), 1–18. 10.1089/ten.2005.11.1 15738657

[B84] Wysocki B. Idaszek J. Buhagiar J. Szlązak K. Brynk T. Kurzydłowski K. J. (2019). The influence of chemical polishing of titanium scaffolds on their mechanical strength and *in-vitro* cell response. Mater. Sci. Eng. C 95, 428–439. 10.1016/j.msec.2018.04.019 30573267

[B85] Xu Y. Li D. Zhu Z. Li L. Jin Y. Ma C. (2020). miR-27a-3p negatively regulates osteogenic differentiation of MC3T3-E1 preosteoblasts by targeting osterix. Mol. Med. Rep. 22 (3), 1717–1726. 10.3892/mmr.2020.11246 32705283 PMC7411295

[B86] Zhang X. Y. Fang G. Xing L. L. Liu W. Zhou J. (2018). Effect of porosity variation strategy on the performance of functionally graded Ti-6Al-4V scaffolds for bone tissue engineering. Mater Des. 157, 523–538. 10.1016/j.matdes.2018.07.064

[B87] Zhao D. Huang Y. Ao Y. Han C. Wang Q. Li Y. (2018). Effect of pore geometry on the fatigue properties and cell affinity of porous titanium scaffolds fabricated by selective laser melting. J. Mech. Behav. Biomed. Mater 88, 478–487. 10.1016/j.jmbbm.2018.08.048 30223211

