# Supplementary material for: The effect of nodal connectivity and strut density within stochastic titanium scaffolds on osteogenesis
Source: Front Bioeng Biotechnol. 2023 Nov 29;11:1305936. doi: 10.3389/fbioe.2023.1305936 (PMC10721980; doi:10.3389/fbioe.2023.1305936)
Supplement: Supplementary file 1 [file DataSheet1.docx]

Supplementary Material

# RT-qPCR reaction methodology

PCR reactions were carried out using a Lightcycler 96 PCR instrument (Roche Diagnostics Ltd, UK). PCR amplification reactions were performed using three technical replicates, in a total volume of 10 μL containing 5 μL 2X GoTaq qPCR mastermix, 0.2 μL 50X GoScript reverse transcriptase mix, final concentrations of 250 nM primers and 2 μL of extracted RNA. Cycling parameters for the RT-qPCR assay were as follows: an initial reverse transcription step at 45 °C for 15 min, reverse transcriptase inactivation/initial denaturation at 95 °C for 2 mins, followed by 45 cycles of 95 °C for 15 sec and an annealing temperature of 60°C for β-actin, osteoponin and osteocalcin or 65°C for collagen for 1 min. A no template control consisting of water instead of RNA, was also added in triplicate.

Primer efficiencies were calculated using serial dilutions of synthetic gblock sequence (Integrated DNA Technologies, USA) and this efficiency was used in ΔΔCt calculations.

# Supplementary Figures


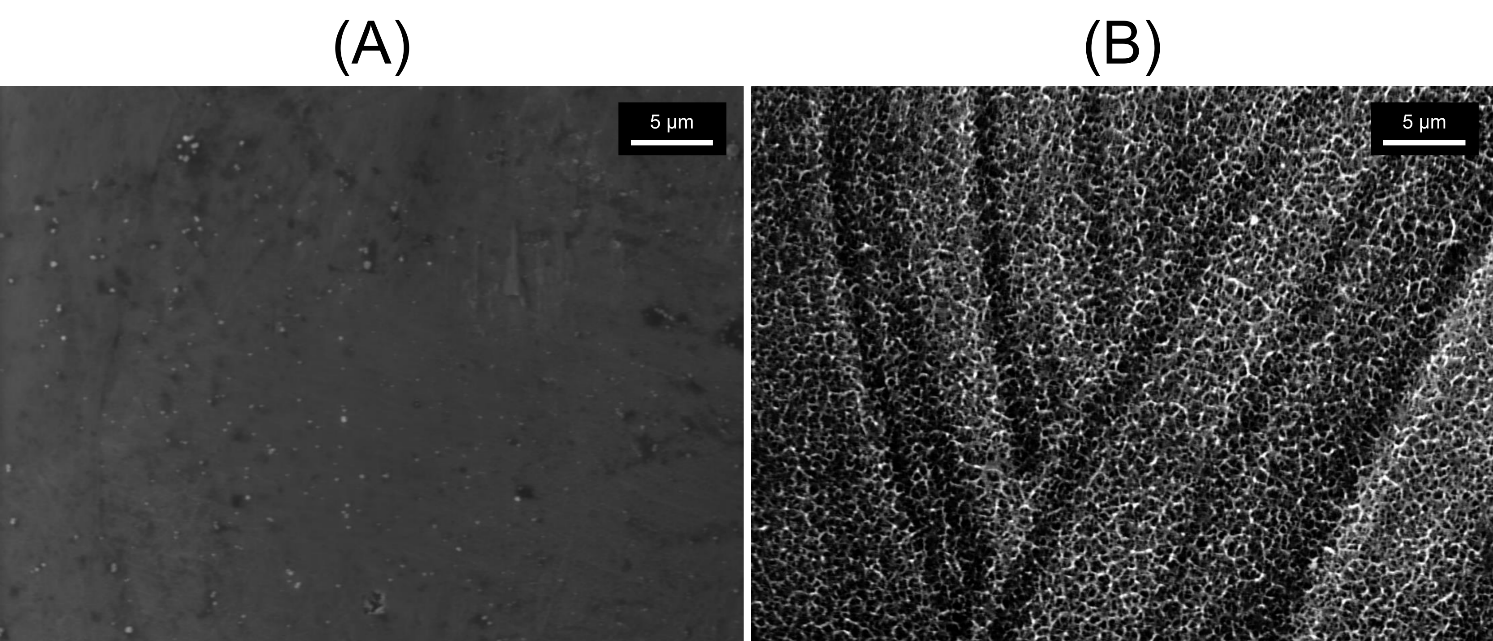


**Supplementary Figure 1**. SEM images demonstrating the different surface topography of additively manufactured pure titanium scaffolds (A) before and (B) after alkali treatment with 4M NaOH.


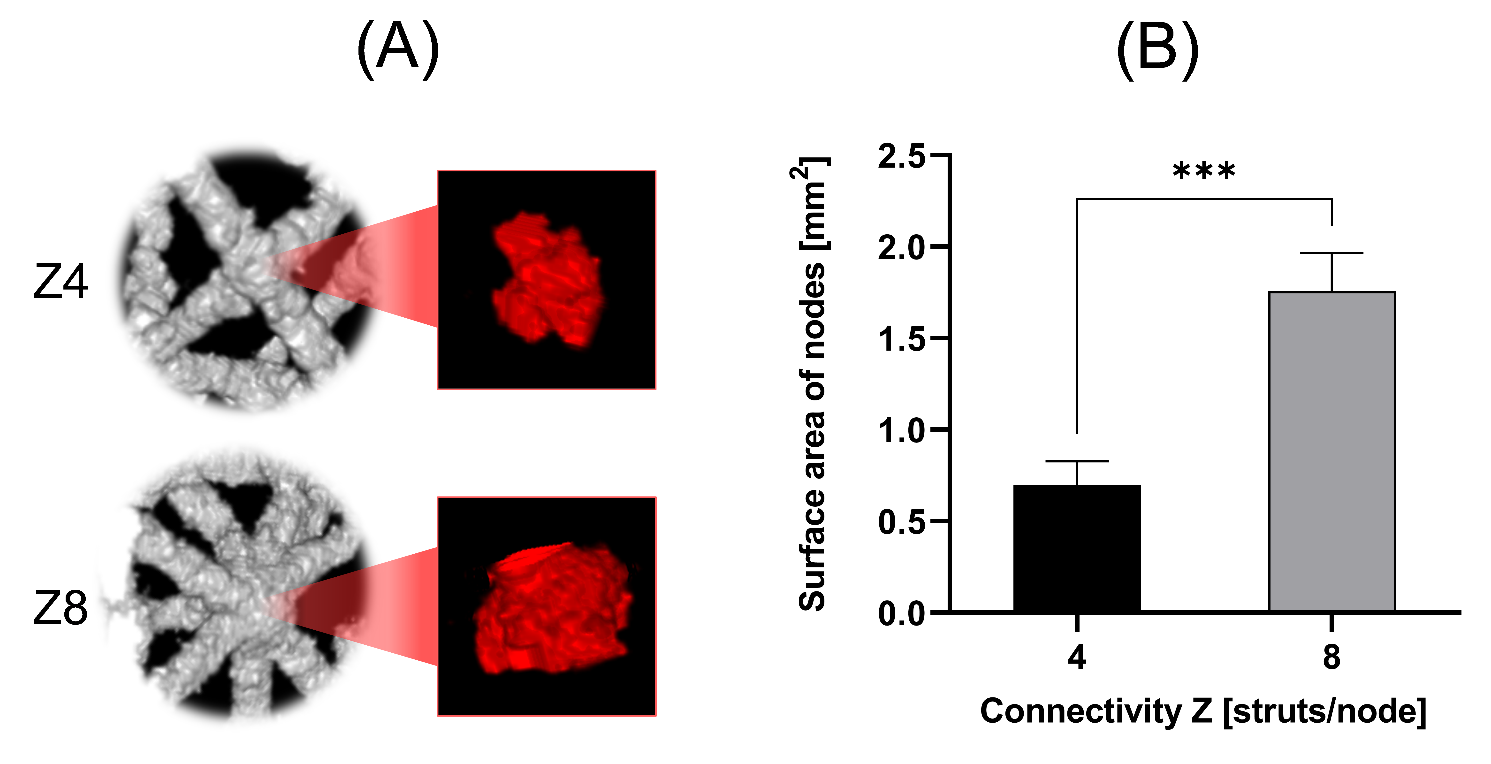


**Supplementary Figure 2**. (A) nodes with 4 and 8 strut connections were isolated from the micro-CT data of scaffolds. (B) the surface area of nodes with connectivity 8 was significantly higher (n=5, p<0.001) than the surface of nodes with connectivity 4.

# Supplementary Tables

**Supplementary Table 1.** Laser parameters used in the additive manufacturing of lattice structures. Layer thickness was 50 μm.

| Strut angle in respect to the building bed | Laser Power | Hatching space [μm] | Exposure time [μs] |
| --- | --- | --- | --- |
| 18º – 36º | 50 | 50 | 321 |
| 36º – 54º | 50 | 50 | 419 |
| 54º – 72º | 50 | 50 | 450 |
| 72º – 90º | 50 | 50 | 438 |

**Supplementary Table 2.** 2-way ANOVA for investigating the effect connectivity Z and strut density d at cell adhesion at 24 hours after seeding (n=3).

| Source of Variation | Degrees of freedom | F-value | P-value |
| --- | --- | --- | --- |
| Z | 1 | F (1, 8) = 0.02192 | 0.10 |
| d | 1 | F (1, 8) = 3.342 | 0.12 |
| Z × d | 1 | F (1, 8) = 3.056 | 0.89 |

**Supplementary Table 3.** 3-way ANOVA for investigating the effect connectivity Z, strut density d and day of culture at proliferation rate (n=3).

| Source of Variation | Degrees of freedom | F-value | P-value |
| --- | --- | --- | --- |
| Day | 4 | F (4, 40) = 4.204 | 0.006 |
| Z | 1 | F (1, 40) = 53.41 | <0.001 |
| d | 1 | F (1, 40) = 64.81 | <0.001 |
| Day × Z | 4 | F (4, 40) = 1.708 | 0.17 |
| Day × d | 4 | F (4, 40) = 1.047 | 0.40 |
| Z × d | 1 | F (1, 40) = 10.45 | 0.002 |
| Day × Z × d | 4 | F (4, 40) = 0.7979 | 0.53 |

**Supplementary Table 4.** 3-way ANOVA for investigating the effect connectivity Z, strut density d and day of culture at the absorbance of picrosirius red (n=3).

| Source of Variation | Degrees of freedom | F-value | P-value |
| --- | --- | --- | --- |
| Day | 1 | F (1, 16) = 304.8 | <0.001 |
| Z | 1 | F (1, 16) = 10.08 | 0.006 |
| d | 1 | F (1, 16) = 24.96 | <0.001 |
| Day × Z | 1 | F (1, 16) = 0.8452 | 0.37 |
| Day × d | 1 | F (1, 16) = 1.479 | 0.24 |
| Z × d | 1 | F (1, 16) = 3.324 | 0.09 |
| Day × Z × d | 1 | F (1, 16) = 6.785 | 0.02 |

**Supplementary Table 5.** 3-way ANOVA for investigating the effect connectivity Z, strut density d and day of culture at the expression levels of ALP (n=3).

| Source of Variation | Degrees of freedom | F-value | P-value |
| --- | --- | --- | --- |
| Day | 3 | F (3, 32) = 204.9 | <0.001 |
| Z | 1 | F (1, 32) = 2.355 | 0.13 |
| d | 1 | F (1, 32) = 43.79 | <0.001 |
| Day × Z | 3 | F (3, 32) = 0.5339 | 0.66 |
| Day × d | 3 | F (3, 32) = 24.36 | <0.001 |
| Z × d | 1 | F (1, 32) = 0.07433 | 0.79 |
| Day × Z × d | 3 | F (3, 32) = 8.686 | <0.001 |

**Supplementary Table 6.** 3-way ANOVA for investigating the effect connectivity Z, strut density d and day of culture at the expression levels of Collagen type-I (n=3).

| Source of Variation | Degrees of freedom | F-value | P-value |
| --- | --- | --- | --- |
| Day | 1 | F (1, 16) = 118.4 | <0.001 |
| Z | 1 | F (1, 16) = 20.67 | <0.001 |
| d | 1 | F (1, 16) = 4.683 | 0.05 |
| Day × Z | 1 | F (1, 16) = 13.85 | 0.002 |
| Day × d | 1 | F (1, 16) = 9.797 | 0.006 |
| Z × d | 1 | F (1, 16) = 0.1340 | 0.72 |
| Day × Z × d | 1 | F (1, 16) = 0.8591 | 0.37 |

**Supplementary Table 7.** 3-way ANOVA for investigating the effect connectivity Z, strut density d and day of culture at the expression levels of Osteopontin (n=3).

| Source of Variation | Degrees of freedom | F-value | P-value |
| --- | --- | --- | --- |
| Day | 1 | F (1, 16) = 219.5 | <0.001 |
| Z | 1 | F (1, 16) = 28.44 | <0.001 |
| d | 1 | F (1, 16) = 0.5199 | 0.48 |
| Day × Z | 1 | F (1, 16) = 26.45 | <0.001 |
| Day × d | 1 | F (1, 16) = 0.4386 | 0.52 |
| Z × d | 1 | F (1, 16) = 1.211 | 0.29 |
| Day × Z × d | 1 | F (1, 16) = 1.162 | 0.30 |

**Supplementary Table 8.** 3-way ANOVA for investigating the effect connectivity Z, strut density d and day of culture at the expression levels of Osteocalcin (n=3).

| Source of Variation | Degrees of freedom | F-value | P-value |
| --- | --- | --- | --- |
| Day | 1 | F (1, 16) = 137.9 | <0.001 |
| Z | 1 | F (1, 16) = 12.61 | 0.003 |
| d | 1 | F (1, 16) = 2.762 | 0.12 |
| Day × Z | 1 | F (1, 16) = 6.644 | 0.02 |
| Day × d | 1 | F (1, 16) = 0.5382 | 0.47 |
| Z × d | 1 | F (1, 16) = 4.990 | 0.04 |
| Day × Z × d | 1 | F (1, 16) = 1.431 | 0.25 |
